# Optical photothermal infrared imaging of fatty acid esterification in the ER of living cells

**DOI:** 10.1126/sciadv.aed6477

**Published:** 2026-07-08

**Authors:** Hannah B. Castillo, Caitlin M. Davis

**Affiliations:** Department of Chemistry, Yale University, New Haven, CT 06511, USA.

## Abstract

Lipotoxicity is an accumulation of lipids that leads to cell death and metabolic disease. Saturated fatty acids are more likely to cause lipotoxicity; however, the mechanism remains unclear due to challenges visualizing reactions in live cells. Here, we use optical photothermal infrared microspectroscopy to investigate palmitic acid (PA) metabolism in hepatocytes with submicron spatial resolution. Upon PA feeding, we found a time-dependent ester carbonyl stretch localized to the endoplasmic reticulum (ER) near lipid droplets with abnormal morphology. This stretch is assigned to diacylglycerol intermediates in the glycerol-3-phosphate pathway. C─D stretches of deuterated PA provide complementary molecular details, supporting a model whereby PA acyl chain packing in the ER reduces enzyme diffusion slowing PA metabolism. Our results provide a deeper understanding of how phase changes induced by high melting temperature fatty acids and their metabolites change ER chemistry as well as provide a tool for detecting chemical and environmental changes associated with lipotoxicity in live cells.

## Teaser

Submicron IR imaging of palmitic acid metabolism in live cells reveals diacylglycerol buildup and gel phase changes in the ER.

## INTRODUCTION

Metabolic diseases such as obesity, diabetes, and metabolic dysfunction-associated steatotic liver disease (MASLD), formerly known as nonalcoholic fatty liver disease, are a major health crisis (*1*). These conditions are widespread; with a worldwide prevalence of more than 30%, MASLD is the most common chronic liver disease (*2*). In these diseases, lipid-induced toxicity, also known as lipotoxicity, plays a major role in their onset and development (*1*). Excess lipid accumulation in the liver, termed steatosis, can lead to even more severe conditions, such as cirrhosis and liver cancer (*1*). A key cause of steatosis is fatty acid (FA) overload, when free fatty acid (FFA) influx and synthesis exceed the liver’s ability to use FFAs as energy or store them in the form of triacylglycerols (TAGs) (*1*, *3*). While FFAs and their metabolites are essential for intracellular signaling and energy homeostasis, an overload of lipids can result in oxidative stress and cell death (*3*). Although these cell stress responses are well documented, due to difficulties directly visualizing metabolic species, the subcellular organization and molecular level details of the underlying chemical conversions that contribute to lipotoxicity are not well understood.

**Fig. 1.**
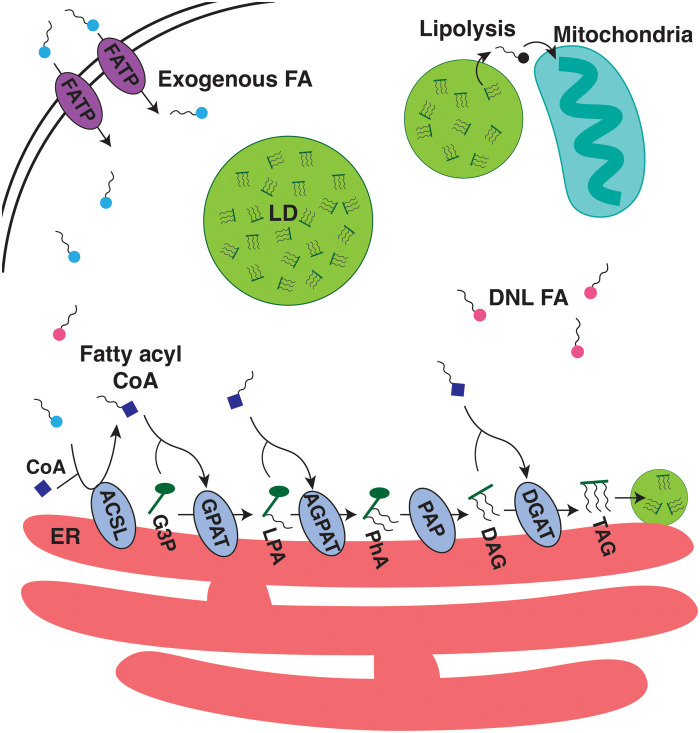
Schematic of TAG synthesis via the G3P pathway. ACSL, Acyl-CoA synthetase; AGPAT, acylglycerol-3-phosphate acyltransferase; DAG, diacylglycerol; DGAT, diacylglycerol transferase; CoA, Coenzyme A; DNL, de novo lipogenesis; ER, endoplasmic reticulum; FA, fatty acid; FATP, fatty acid transporter protein; G3P, glycerol-3-phosphate; GPAT, G3P acyltransferase; LD, lipid droplet; LPA, lysophosphatidic acid; PAP, phosphatidate phosphohydrolase; PhA, phosphatidic acid; TAG, triacylglycerol.

Lipid droplets (LDs) protect against lipotoxicity, serving as depots for neutral lipids. Ubiquitous in cells, LDs are composed of a core of neutral lipids bound by a phospholipid monolayer (*4*, *5*). Once regarded simply as storage pools of lipids, recent work has shown that LDs are dynamic organelle that regulate the flux of lipids through periods of growth and consumption (*4*). This process closely reflects the metabolic state of the cell, as the LD will sequester potentially toxic lipids in periods of FA overload and release them when the cell is in need of energy (*5*). LDs are closely associated with the endoplasmic reticulum (ER), where most of the hepatic TAGs are synthesized via the glycerol-3-phosphate (G3P) pathway (Fig. 1) (*4*, *6*). Precursors to this pathway include both exogenous FAs taken up by the cell through diffusion or fatty acid transporter proteins as well as de novo lipogenesis (DNL)–derived FAs. Once in the cell, FAs must be activated by conjugation to coenzyme A (CoA) via long-chain acyl-CoA synthetases, forming fatty acyl-CoA molecules (*6*). These molecules then undergo stepwise esterification to G3P by multiple enzymes in the ER membrane, ultimately forming TAGs (*7*–*9*). TAGs are deposited within the leaflets of the ER bilayer until they reach a critical concentration, resulting in phase separation and coalescing into a lipid lens that expands and eventually buds from the ER membrane in the form of a LD (*4*, *5*, *10*). While LDs serve as an excellent defense against lipotoxicity, increased levels of exogenous FAs can still lead to steatosis and have been shown to alter this carefully regulated pathway (*11*).

Furthermore, exogenously supplied saturated and unsaturated FAs have differential effects on cells. Excess levels of palmitic acid (PA), a saturated FA, induce inflammation, ER stress, and, ultimately, cell death (*12*–*14*). Conversely, excess oleic acid (OA), an unsaturated FA, while inducing steatosis, does not cause the same toxic effects and even suppresses PA-induced apoptosis in cells (*13*, *14*). These effects are also evidenced in the resulting LD morphology. While both FAs cause an increase in LD size in comparison to control conditions, LDs resulting from OA feeding are often bigger than LDs in PA-fed cells. Meanwhile, PA-feeding causes a larger increase in the number of LDs per cell than OA feeding (*14*, *15*). This suggests that PA and OA trigger a spatial regulation of lipid metabolism involving LD morphology. While it is known that various FAs conversely contribute to cell fates and liver disease, missing is a mechanistic understanding of how these disruptors affect localized metabolism at the subcellular level. Several theories have emerged. One theory is that PA’s toxicity is due to its lower incorporation into LDs, changing the ratio of esterified to toxic unesterified FAs (*13*, *16*). Others attribute PA’s toxicity to down-regulation of TAG synthesis enzymes contributing to metabolic dysfunction (*17*). Thus, investigating PA localization and metabolism at a subcellular level is essential for understanding its role in disease.

**Fig. 2.**
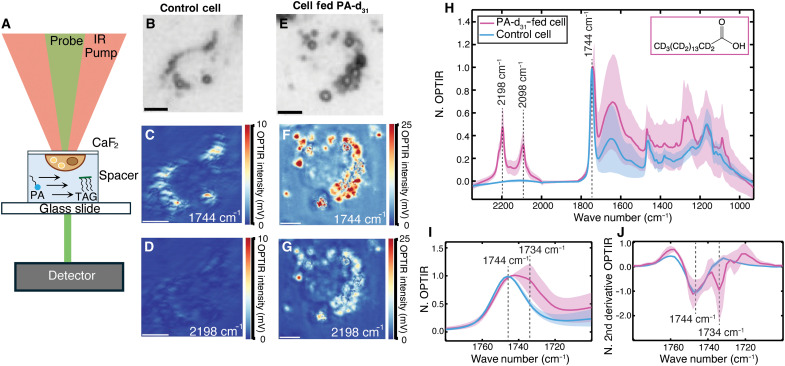
Huh-7 cells fed PA-d_31_ exhibit an unanticipated shoulder off the C═O ester carbonyl stretch of TAGs/CEs. (**A**) Schematic of live-cell imaging using OPTIR imaging. CaF_2_, calcium fluoride; IR, infrared. (**B**) Bright-field image of a live control Huh-7 cell. (**C**) Single wave number image of the live control cell collected at the C═O lipid carbonyl band (1744 cm^−1^). (**D**) Single wave number image of the live control cell collected at the *v*_as_ C─D_2_ stretch (2198 cm^−1^). (**E**) Bright-field image of a live Huh-7 cell 24 hours after feeding 60 μM PA-d_31_ conjugated to BSA at a 2:1 ratio. (**F**) Single wave number image of the live PA-d_31_–fed cell collected at the C═O lipid carbonyl band (1744 cm^−1^). (**G**) Single wave number image of the live PA-d_31_–fed cell collected at the *v*_as_ C─D_2_ stretching mode (2198 cm^−1^). (**H**) Average of 20 normalized OPTIR spectra collected across 20 LDs of four control Huh-7 cells (blue) and average of 20 normalized OPTIR spectra collected across 20 LDs of four Huh-7 cells 24 hours after feeding 60 μM PA-d_31_ conjugated to BSA at a 2:1 ratio (pink). Figure displays four biological replicates and 20 technical replicates. Shading is one SD of the average normalized spectrum. The dominant C═O peak at 1744 cm^−1^, attributed to the ester carbonyl of TAGs/CEs, confirms that both cells are imaged on LDs. Only the cell fed PA-d_31_ exhibits C─D_2_ asymmetric and symmetric stretches at 2198 and 2098 cm^−1^, respectively. (**I**) A close-up of the C═O spectral region from 1700 to 1780 cm^−1^. A shoulder at 1734 cm^−1^ is observed only in PA-d_31_–fed cells. (**J**) Second derivative of the C═O region resolves the shoulder at 1734 cm^−1^.

Here, we leverage the submicron resolution of optical photothermal infrared (OPTIR) imaging to track PA metabolism in live hepatocytes (Fig. 2A). We have previously used OPTIR to track DNL and to monitor the effect of OA on DNL (*11*, *18*). In this work, we feed deuterated palmitic acid (PA-d_31_) to the hepatocyte-derived cell line Huh-7 and use C─D stretches and ester carbonyl shifts to monitor FA uptake, metabolism, and incorporation into LDs. Our results support both the theory that PA alters storage of FAs in LDs and down-regulates TAG synthesis. Compared to OA, we observe slowed PA metabolism in the ER near LDs with abnormal morphology. We find that the two effects are coupled to phase changes in the ER that induce ER stress. Using the C─D stretches of PA-d_31_, we demonstrate that upon PA feeding, TAG precursors in the G3P pathway enter a rigid gel phase by tightly packing their acyl chains. Using the C═O stretch of PA-d_31_, we assign the TAG precursor to diacylglycerol (DAG). We conclude that acyl chain packing slows metabolism, leading to the buildup of DAGs in the ER, which in turn inhibits the ability of LDs to properly emerge from the ER, giving them an abnormal morphology. Azido PA, which packs less tightly than PA due to the azide, did not exhibit any of these effects. Similarly, unsaturated FAs like OA that have lower melting temperatures have been found to rescue cells from PA-induced apoptosis. Overall, this work sheds light on the toxic origins of PA and other saturated FAs, as well as establishes tools for understanding PA chemistry in the ER and the function of LDs in lipotoxicity.

## RESULTS

### PA feeding reveals an unanticipated spectral feature at 1734 cm^−1^

To observe how PA is taken up and stored by the cell, we fed cells 60 μM PA-d_31_ conjugated to bovine serum albumin (BSA) at a 2:1 ratio, in line with physiological FFA concentrations that fluctuate from submillimolar to millimolar (*19*, *20*). Cells were fed PA-d_31_ once at time 0 and were not refed throughout the duration of the experiments. Deuteration of the C─H bonds in PA shifts the frequency of the asymmetric and symmetric C─H_2_ stretching modes from 2916 and 2850 to 2198 and 2098 cm^−1^, respectively (fig. S1). These C─D_2_ stretches lie in the “cell silent region,” enabling detection of exogenously obtained PA without interfering signals from endogenously synthesized PA or other cellular molecules. The deuteration does not alter the structure of PA, and therefore, it accurately mimics PA. Nor does the deuteration of PA affect cell viability at physiological FFA concentrations (fig. S2, A to C). Further, the carbon chains are preserved as PA is metabolized, primarily through esterification and storage in LDs, allowing assignment of any species with these peaks as a derivative of PA-d_31_. Free PA is generally not observable outside of LDs due to the detection limit of the instrument (fig. S3, A to F), in line with reported detection limits of similar instruments (*11*, *21*).

As most FAs end up esterified in LDs, initial spectra were collected on LDs of live Huh-7 cells 24 hours after feeding. Single wave number images were collected at frequencies attributed to deuterated lipids to confirm the uptake of PA-d_31_. Control cells only fed BSA (vehicle control; Fig. 2B) only show signal in infrared (IR) images collected at 1744 cm^−1^, originating from the ester carbonyl stretch of TAGs and cholesteryl esters (CEs) in the LDs (Fig. 2C) (*22*). No signal is seen in IR images collected at 2198 cm^−1^, the asymmetric C─D_2_ stretch that would indicate a deuterated lipid (Fig. 2D). Cells fed PA-d_31_ (Fig. 2E) show signal in IR images collected at both 1744 and 2198 cm^−1^ that overlap well with each other as well as the LDs in the brightfield, indicating that the PA-d_31_ has been taken up by the cell, esterified, and stored in LDs (Fig. 2, F and G). This is also confirmed by the strong C─D stretches in single spectra collected on LDs of cells fed PA-d_31_ (Fig. 2H). A 1734-cm^−1^ shoulder off this ester carbonyl peak (Fig. 2I) was observed in numerous PA-fed spectra. A second derivative further resolves this peak from the nearby 1744-cm^−1^ band (Fig. 2J). This spectral feature is only found in lipid-rich regions of cells fed PA-d_31_ and is always associated with C─D stretches, suggesting that it is a derivative of PA and therefore a lipid. To confirm that the shoulder is not an optical artifact derived from the deuterium labels on the PA, unlabeled PA was fed to cells at the same concentration, and the same shoulder was observed (fig. S4, A and B).

### Timelapse of the 1734-cm^−1^ peak suggests a metabolic intermediate

We hypothesize that the 1734-cm^−1^ shoulder arises from a metabolic intermediate of PA in the G3P pathway that generates TAGs. If true, there would be a temporal component to the C═O stretch as the PA-d_31_ is processed and not refed. To test this hypothesis, Huh-7 cells were incubated with PA-d_31_ and monitored by OPTIR approximately every 3 hours for 70 hours. An average of 77 spectra per time point were collected in LDs from approximately six cells per time point (total *n* = 133), and each cell’s corresponding spectra were averaged. Spectra were collected by line scans with a spacing of ~300 to 500 nm or individual points on multiple LDs. This revealed a temporal trend to the 1734-cm^−1^ shoulder.

**Fig. 3.**
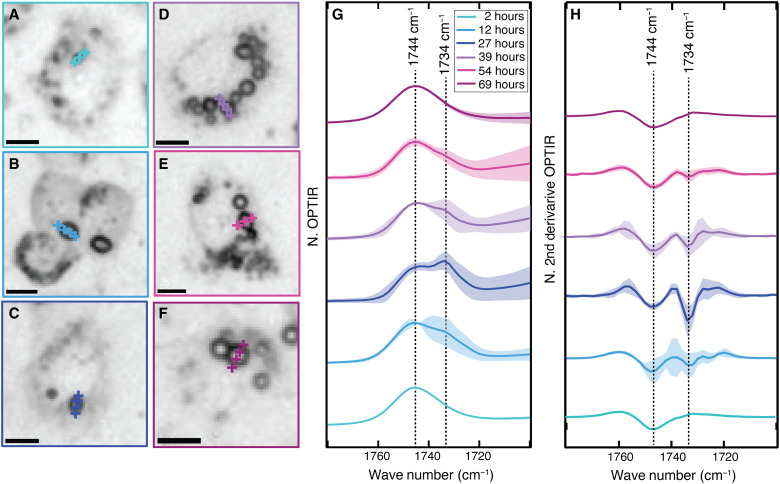
Timelapse of the ester carbonyl stretch region from 2 to 70 hours postfeeding 60 μM PA-d_31_ conjugated to BSA at a 2:1 ratio illustrated by spectra from cells representing the average data with 1734-cm^−1^ shoulder. (**A** to **F**) Bright-field images of representative Huh-7 cells fed at 2, 12, 27, 39, 54, and 69 hours, respectively. Colored crosses indicate line scans where corresponding spectra were collected and are plotted in (G). Scale bars, 10 μm. (**G**) Average normalized IR spectra with SD of Huh-7 cells fed PA-d_31_ at 2, 12, 27, 39, 54, and 69 hours. Each time point represents the average spectra from the points labeled in (A) to (F). Shading is one SD of the average normalized spectrum. Figure displays 1 biological replicate and an average of 25 technical replicates per time point. (**H**) Second derivative of the average spectra in (G) with corresponding SDs.

Throughout 72 hours, the LDs of cells grew with increasing time (Fig. 3, A to F). For the first 12 hours, the 1744-cm^−1^ ester carbonyl peak of TAGs/CEs dominates the lipid region in the spectra. The peak at 1734 cm^−1^ first emerges as a small shoulder off the 1744-cm^−1^ peak around 12 hours after initial feeding (Fig. 3G). There was cell-to-cell heterogeneity in the exact time postfeeding that the shoulder presented, consistent with our past work that lipid metabolism is highly heterogeneous between cells (*11*, *18*). Over the next few hours, the intensity of the 1734-cm^−1^ band slowly increased until it was the dominating species in the ester carbonyl region. Between 27 and 54 hours, the 1734-cm^−1^ peak was visible in almost all investigated cells. After which, it slowly merges back into the 1744-cm^−1^ peak between 52 and 70 hours after feeding. By 69 hours after feeding, the 1734-cm^−1^ peak is undetectable Fig. 3G). Averaged spectra from all cells in each time point in this experiment show the same trend (fig. S5A). Taking the second derivative of the averaged spectra confirms this trend (Fig. 3H), with the 1734-cm^−1^ peak appearing around 12 hours postfeeding, growing and predominating over the 1744-cm^−1^ stretch and shrinking until it is indistinguishable from the 1744-cm^−1^ TAG peak by 69 hours postfeeding. This is also confirmed by taking the second derivative of the averaged spectra of all cells in each time point of this experiment (fig. S5B). In addition, the distribution of 1734-cm^−1^ intensity of all spectra in each time point shows the spread of increasing and decreasing 1734-cm^−1^ intensity throughout the time (fig. S5C). These data are consistent with our hypothesis that the unknown lipid peak is a metabolic intermediate that is built up to a concentration detectable via OPTIR over the first 27 hours and then processed to a concentration undetectable via OPTIR over the following 42 hours. Further, from 12 to 27 hours, the second derivative reveals additional peaks at 1726 and 1738 cm ^−1^ that indicate low populations of other metabolic intermediates (Fig. 3H).

### Intermediate buildup occurs in the ER

**Fig. 4.**
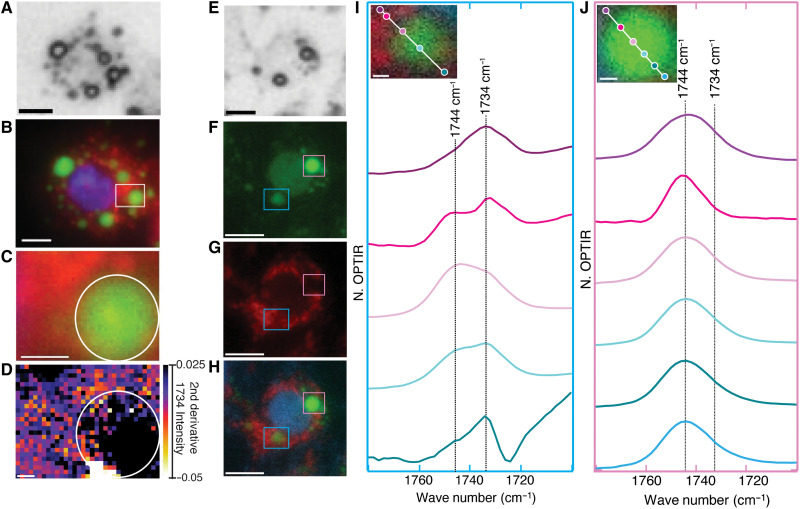
PA intermediates buildup in the ER. (**A**) Bright-field image of a cell 24 hours after feeding 120 μM PA-d_31_ conjugated to BSA at a 2:1 ratio. Scale bar, 10 μm. (**B**) Fluorescence image of the cell in (A) stained for the ER (red), nucleus (blue), and LDs (green). A white box indicates the hyperspectral image bounds. Scale bar, 10 μm. (**C**) Fluorescence signal in the hyperspectral image area with the LD outlined in white. Scale bar, 1 μm. (**D**) Single wave number image of the second derivative of the 1734-cm^−1^ band in the hyperspectral image with the LD outlined in white. (**E**) Bright-field image of a cell 24 hours after feeding 120 μM PA-d_31_ conjugated to BSA at a 2:1 ratio. (**F**) The green fluorescence channel of the cell in (E) stained for LD. Pink and blue boxes indicate the studied LDs. Scale bar, 10 μm. (**G**) The red fluorescence channel of the cell in (E) stained for ER. Pink and blue boxes indicate the studied LDs. Scale bar, 10 μm. (**H**) The cell in (E) stained for the ER (red), LD (green), and nucleus (blue). Pink and blue boxes indicate the studied LDs. Scale bar, 10 μm. (**I**) Spectra corresponding to the points where a line scan was collected across the LD in the blue box (inset). Lipid bands at 1744 and 1734 cm^−1^ are marked. Scale bar, 1 μm. (**J**) Spectra corresponding to the points where a line scan was collected across the LD in the pink box (inset). Lipid bands at 1744 and 1734 cm^−1^ are marked. Scale bar, 1 μm. All images collected on the OPTIR microscope.

Bright-field images reveal changes in the morphology of cells fed PA-d_31_. LDs in cells fed PA-d_31_ increased in both size and number compared to control cells (Fig. 4A). This observation agrees with other studies that report PA induces an increase in LD size and number (*14*, *23*). In addition to this, LD morphology is altered; many LDs in PA-fed cells are oblong shape (fig. S6, A and B). LD morphology was quantified in 50 LDs from control or PA-fed cells, consistent with our qualitative observations the ellipticity of PA-fed cells is 0.8 ± 0.1 and control cells is 0.94 ± 0.06 (table S1). These oblong LDs tend to have strong 1734-cm^−1^ signal and are only observed when feeding PA, not in control conditions nor when feeding unsaturated FAs such as OA (fig. S6, C to F). Abnormal LD morphology in combination with the hypothesis that the 1734-cm^−1^ intermediate species is from TAG synthesis suggests that the buildup of these intermediates affects how LDs bud from the ER.

LDs have many contact points with other organelle, including the ER, mitochondria, and peroxisomes (*4*, *24*). These contact points form metabolic hubs imperative to FA homeostasis and facilitate FA trafficking, contributing to LD growth and degradation (*4*, *24*). We focus on the ER because TAG synthesis and LD nucleation occur in the ER. ER-LD contact sites are typically 10 to 80 nm, which are below the sub-500-nm spatial resolution of the OPTIR (*25*). Nevertheless, OPTIR can test our hypothesis that intermediate buildup in the ER affects how LDs bud from the ER; OPTIR can resolve whether the intermediate occurs in LDs colocalized with the ER, whether it is isolated to the ER or LD, and whether the LD has abnormal morphology. As we believe that the 1734-cm^−1^ peak is a PA-d_31_ metabolite, it should increase in intensity when the concentration of PA-d_31_ fed to cells is increased. Thus, to better resolve the shoulder, Huh-7 cells were incubated with PA-d_31_ at a slightly higher physiological concentration of 120 μM conjugated to BSA at a 2:1 ratio. After 24 hours, cells were fluorescently stained for the nucleus, ER, and LD with Hoechst nuclear stain (blue), ER-Red (red), and LipiSpot (green), respectively. Cells were then imaged live on the OPTIR. Cells with multiple, large LDs were chosen for analysis (Fig. 4A).

To determine whether the 1734-cm^−1^ peak is associated with the ER and/or spatially confined to the LDs, hyperspectral images were collected across LDs in regions with high ER fluorescence (Fig. 4, B to D). Hyperspectral maps are three-dimensional images composed of OPTIR spectra from 1600 to 2348 cm^−1^ collected at every pixel within the area of interest. From a hyperspectral map, a single wave number image of the second derivative of the 1734-cm^−1^ peak reveals the spatial distribution of the metabolic intermediate relative to TAGs/CEs. Higher 1734/1744 cm^−1^ ratios are found both at the edge of LDs bordering the ER and in the ER itself (Fig. 4D). This indicates that the metabolic intermediate is concentrated in the ER and likely near LD-ER contact sites. Still, to rule out that the 1734-cm^−1^ peak originates from the ER itself rather than a metabolic intermediate, a control cell was stained for the ER, nucleus, and LD and imaged live on the OPTIR (fig. S7, A and B). 1744 cm^−1^ is only detected at the center and edge of the LD, and only amide I and water signal are present in the ER (fig. S7C).

To further confirm that the intermediate is arising from chemistry in the ER, a cell with both a LD in an ER-rich region and a LD in an ER-free region was investigated (Fig. 4E). This was confirmed by separating the red, green, and blue fluorescence channels to identify LDs with high overlap with the ER (Fig. 4, F to H). Line scans were collected over LDs with high or low ER overlap. (Fig. 4, I and J). Like the droplet in Fig. 4A, the LD with potential to make ER contacts had regions of increased 1734-cm^−1^ intensity at the LD edge bordering the ER. At the edges of the LD, the 1734-cm^−1^ band dominates, while the 1744-cm^−1^ band dominates in the center of the LD (Fig. 4I; full spectra in fig. S8A). The 1734-cm^−1^ peak persisting throughout the center of the LD likely originates from metabolic intermediates in the ER surrounding the LD, as there is high red fluorescence overlap with this LD. In contrast, the LD in an area without potential to make ER contacts (Fig. 4H) did not have any regions with strong 1734-cm^−1^ signal (Fig. 4J; full spectra in fig. S8B), indicating that there are no metabolic intermediates building up in or around that LD. This LD also did not have high fluorescence overlap, indicating that it is further from the ER where intermediates accumulate. This further highlights the heterogeneity of the LDs within cells, as some exhibit this 1734-cm^−1^ lipid species and some do not. This trend was confirmed through complementary fluorescence and IR analysis of a total of eight LDs; only LDs in ER-rich regions have the 1734-cm^−1^ signal and thus metabolic intermediate buildup (fig. S9, A to P). As TAG synthesis occurs in the ER and LDs are known to have multiple contacts with the ER even after budding, we hypothesize that the 1734-cm^−1^ lipid species is a precursor of TAG synthesis arising from PA feeding.

### DAGs accumulate in Huh-7 cells upon PA feeding

Previous work has shown that lipid metabolism is affected by PA feeding (*17*, *26*, *27*). Cells fed PA tend to have large increases of glycerolipids in comparison to control conditions. Specifically, lipodomics find that large amounts of disaturated DAGs accumulate (*26*, *27*). We hypothesize that the 1734-cm^−1^ lipid species is a disaturated DAG building up to a concentration that is observable by OPTIR.

**Fig. 5.**
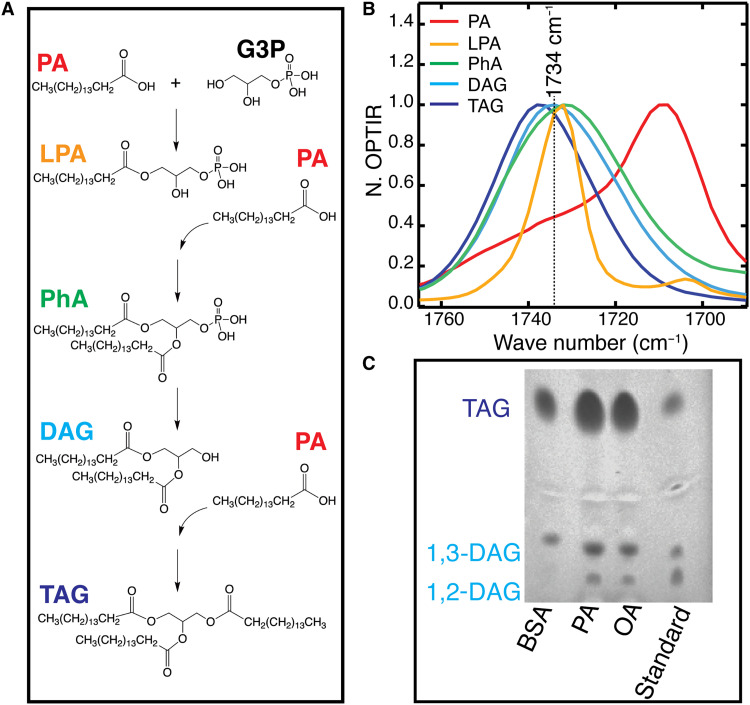
Assignment of 1734 cm^−1^ to DAGs. (**A**) TAG synthesis via the G3P pathway with PA as the FA chains. (**B**) OPTIR spectra of TAG intermediates dissolved in chloroform at 25 mM. Figure displays one technical replicate. (**C**) Thin layer chromatography (TLC) plate of lipids isolated from PA-fed, OA-fed, and control cells. Cells were fed 60 μM BSA-conjugated PA or OA or BSA alone (control) and incubated for 24 hours. Tripalmitin (TAG), 1,3 dipalmitin (1,3-DAG), and 1,2 dipalmitin (1,2-DAG) were used as standards. Figure displays one biological replicate and one technical replicate.

TAG synthesis by the G3P pathway includes five primary lipid species: an FFA, lysophosphatidic acid (LPA), phosphatidic acid (PhA), DAG, and TAG (Fig. 5A). To assign the 1734-cm^−1^ peak, we compare it to the OPTIR spectra of these five lipid species. As we established that the unassigned dominant intermediate arises from PA feeding, the acyl chains of all TAG precursors analyzed were solely composed of PA. IR spectra for PA, palmitoyl lysophosphatidic acid, 1,2- dipalmitoyl-*sn*-glycero-3-phosphate, 1,2- dipalmitoyl-*sn*-glycerol, and tripalmitin were collected on the OPTIR. However, the vibrational frequency of a bond, especially carbonyls, is dependent on the local electric field as well as nearby hydrogen bonds (*28*). Thus, the spectrum of each lipid species may differ slightly depending on the solvent. Previous work has shown that the ER has a polarity between chloroform and dichloromethane (DCM) (*29*). Therefore, to replicate the polarity of the ER, all five precursors were dissolved in chloroform at a concentration of 25 mM (Fig. 5B). The solubility of PhA and LPA in DCM was below the detection limit of the OPTIR; therefore, only PA, DAG, and TAG were compared in DCM (fig. S10). Comparing the OPTIR spectra in chloroform, the carboxylic acid carbonyl of PA is at 1709 cm^−1^, while the rest of the precursors have ester carbonyls with peaks that are in the range of 1730 to 1740 cm^−1^. As anticipated, the bandwidth and peak position of the ester carbonyl of DAGs in chloroform best match the in cellulo peak at 1734 cm^−1^. In DCM, DAG appears at 1736 cm^−1^, also closely matching the 1734-cm^−1^ peak. Thus, we assign the 1734-cm^−1^ peak to DAGs.

However, because C═O stretches are sensitive to the environment, we questioned whether the 1734-cm^−1^ shoulder might arise from TAG in the differing environments of the ER and LDs. To test this, cells were fed 120 μM ^13^C-labeled PA conjugated to BSA at a 2:1 ratio and imaged live 24 hours later. The heavier carbon isotope of the ^13^C═O causes a redshift in the lipid ester carbonyl stretch, shifting the ester carbonyl of all lipid species derived from ^13^C PA (*30*). If both the ^13^C-labeled peak and the ^12^C═O TAG peak have a shoulder in the ER, this would confirm that the shoulder is due to two TAG species in different environments. However, if the shoulder appears only on the ^13^C═O carbonyl peak, then it would be confirmation that it is a lipid species derived from ^13^C PA. As expected, a spectrum collected in the ER exhibited a redshifted ester carbonyl band at 1700 cm^−1^ (fig. S11). No shoulder appears on the ^12^C═O band. A small shoulder off the ^13^C═O carbonyl is located at 1686 cm^−1^, consistent with the DAG intermediate building up in the ER.

To further confirm the DAG assignment, the cell’s lipid profile was assessed using thin layer chromatography (TLC). Cells were incubated with 60 μM BSA-conjugated PA or OA or BSA alone (vehicle control) for 24 hours. Lipids were then isolated from the cell using the Bligh and Dyer lipid extraction technique and spotted onto TLC plates (Fig. 5C). In comparison to control cells fed BSA, PA- and OA-fed cells both produced large amounts of TAGs and 1,2-DAGs. This partially agrees with a previous study that used TLC to separate lipid extracts from cardiomyoblasts; however, in cardiomyoblasts, PA led to the formation of substantially more DAGs than OA (*31*). The TLC plate was quantified, and PA-fed cells had 4.4% of the 1,2-DAGs in the total lipid profile, while OA-fed cells had 3.1% of 1,2-DAGs in the lipid profile, resulting in 1.4 more 1,2-DAGs in the PA-fed cells than in OA-fed cells. In hepatocytes, the increase in DAGs upon PA and OA feeding is not as drastic (Fig. 5C). This is interesting as DAGs are only easily detected using OPTIR on PA-fed hepatocyte cells, suggesting that they are more evenly distributed across the ER (below the detection limit) of OA-fed hepatocyte cells. We previously used the same OPTIR approach to monitoring OA-d_33_ feeding in hepatocytes and adipocytes, and no such TAG precursor peak had been observed (*11*). To further investigate, Huh-7 cells were fed 60 μM OA-d_33_ conjugated to BSA at a 2:1 ratio to match the PA time-dependent study. Cells fed OA-d_33_ were then studied using OPTIR throughout 24 to 48 hours. Line scans were collected through large LDs. Unexpectedly, a few LDs exhibited a shoulder at 1738 cm^−1^ off the 1744-cm^−1^ carbonyl stretch (fig. S12A). Although this peak was found in OA-fed cells, it was far less common (fig. S12B). Furthermore, no LDs with abnormal morphology were observed (fig. S12, C and D). This suggests that similar amounts of TAGs and DAGs are present in both PA- and OA-fed Huh-7 cells, but their spatial distributions and cell phenotypes differ. That DAGs are infrequently detected by OPTIR in OA-fed Huh-7 cells indicates that they are distributed across the cell such that the local concentration is below the detection limit of the OPTIR. Thus, another factor exists that is contributing to the large local buildup of DAGs in the ER of PA-fed Huh-7 cells.

### ER rigidity is correlated with metabolic inhibition

A physical difference between OA and PA is their melting temperature. In general, saturated fats have higher melting temperatures than unsaturated fats because their straight acyl chains can be tightly packed. The melting temperature of OA is ~13°C, while the melting temperature of PA is ~63°C (*32*). This trend is maintained through the five primary lipid species in TAG synthesis; the melting temperature of triolein is ~5°C, while the melting temperature of tripalmitin is ~45° to 66°C (*32*, *33*). Thus, at physiological temperatures, OA and its TAG intermediates are a liquid, whereas PA and its TAG intermediates are a solid. Mixtures of FAs have lower melting temperatures than pure FA (*34*); however, FA feeding artificially inflates the cellular distribution of the fed FA. We therefore hypothesize that PA and its TAG intermediates solidify in the ER, leading to ER stress and DAG accumulation.

To test this hypothesis, we investigate the C─D stretches of the acyl chains. As described above, differences in solvent polarity were considered in the assignment of the shifted lipid carbonyl peak. However, other physical properties of lipids can alter vibrational spectra, most notably the conformation and structure of the lipid (*35*–*37*). Lipids are polymorphic and, under physiological conditions, can undergo a lamellar gel to lamellar liquid-crystalline phase transition. The lamellar gel state is characterized by fully extended and rigid acyl chains in an all-trans conformation, resulting in ordered and tightly packed lipids (*35*). In contrast, the lamellar liquid-crystalline state is a disordered environment, with more hydrocarbon chains in the gauche conformation, allowing for increased dynamics of lipid molecules (*35*). Previous studies have demonstrated that the C─H vibrational modes can distinguish between these two states (*38*, *39*). The liquid crystalline phase is characterized by higher frequencies and broadening of the CH_2_ bands due to the increase in dynamics between acyl chains.

**Fig. 6.**
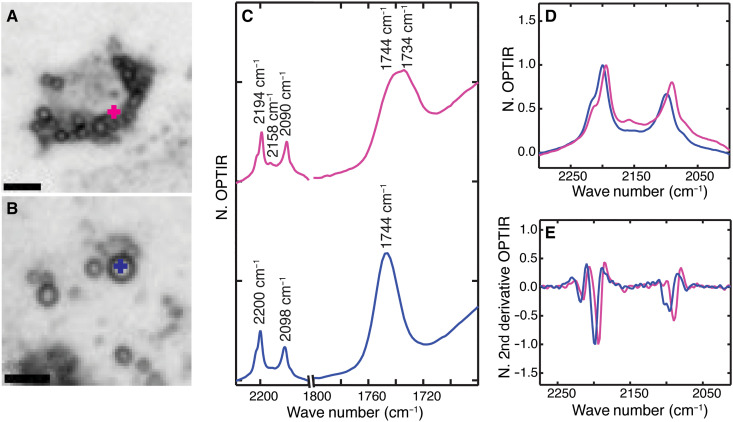
The 1734-cm^−1^ signal appears in an ordered lamellar gel environment. (**A**) Bright-field image of a Huh-7 cell fed 60 μM PA-d_31_ conjugated to BSA at a 2:1 ratio. The spectrum was collected as a single spectrum on the edge of a LD near the ER 24 hours after being fed PA-d_31_ (indicated by the pink crosshairs). (**B**) Bright-field image of a Huh-7 cell fed 60 μM PA-d_31_ conjugated to BSA at a 2:1 ratio. The spectrum was collected as a single spectrum on the center of a LD of a cell 69 hours after being fed PA-d_31_ (blue crosshairs). (**C**) A redshift of the asymmetric and symmetric CD_2_ stretches and the appearance of a Fermi resonance at 2158 cm^−1^ are correlated with the appearance of a 1734-cm^−1^ shoulder off the lipid carbonyl. Enlarged view of the C─D region and its corresponding second derivative spectra is shown in (**D**) and (**E**), respectively. Measurement of multiple cells and samples produced the same results (fig. S8). Spectra were normalized by prominent peak heights in the C─D region. Figure displays one biological and one technical replicate.

The C─D vibrational modes of Huh-7 cells were imaged by OPTIR 24 hours after PA-d_31_ feeding. There is a stark difference between the symmetric and asymmetric CD_2_ stretches in spectra with and without the 1734-cm^−1^ lipid carbonyl peak. In an OPTIR spectrum taken on a LD far from the ER, the 1744-cm^−1^ lipid carbonyl dominates. Consistent with a liquid-crystalline phase, the corresponding symmetric and asymmetric CD_2_ stretches are located at 2098 and 2200 cm^−1^, respectively (Fig. 6A, bottom). These are TAGs made with PA-d_31_ acyl chains esterified and stored in LDs. When focused on the edge of a LD near the ER, the 1734-cm^−1^ peak dominates the lipid carbonyl region, and the symmetric and asymmetric CD_2_ stretches redshift to 2090 and 2194 cm^−1^, respectively (Fig. 6A, top). This is consistent with PA-d_31_ acyl chains in a lamellar gel phase. Further, a small peak appears at 2158 cm^−1^, which can be assigned as Fermi resonance between the symmetric CD_2_ mode and the first overtone of the CD_2_ bending mode (Fig. 6B) (*38*, *40*, *41*). Fermi resonant interactions are sensitive to conformational changes of a molecule and have been used to monitor intermolecular interactions (*42*–*44*). This particular Fermi resonance has been reported to disappear during the lipid transition from the ordered lamellar gel phase to the liquid crystalline phase (*38*, *45*). Second derivative spectra confirm the appearance of the Fermi resonance as well as the shifting of the CD_2_ peaks (Fig. 6C). These results are reproducible across multiple spectra with and without the 1734-cm^−1^ peak. In most cases, the dominance of the 1734-cm^−1^ peak in the lipid carbonyl region is associated with a CD_2_ redshift and the appearance of the Fermi resonance. As previously established, these spectral features are most common at the edges of a LD colocalized with the ER. Thus, as hypothesized, we conclude that it is lipids in the ER that are forming a lamellar gel state due to the incubation of PA which creates tighter packing of the acyl chains of TAG precursors in the ER. Most biomembranes, including the ER membrane, exist in the liquid crystalline state to allow for diffusion of biomolecules (*46*). We further speculate that enzymatic reactions necessary for TAG synthesis are diffusion limited in the lamellar gel state, slowing PA metabolism. To investigate this, fluorescence recovery after photobleaching (FRAP) was performed on control cells and cells fed 120 μM PA-d_31_ using an ER-mEGFP plasmid that localizes to the ER. The fluorescence recovery of control and PA-fed cells was similar. We determined diffusion times from the resulting FRAP curves and identified a diffusion time of 3 ± 1 s for control cells and diffusion time of 8 ± 1 s for cells fed PA-d_31_ (fig. S13). This further confirms that metabolites building up in the ER promote ER rigidity and affect enzyme mobility.

In addition to C─D stretches, azides are particularly sensitive to local environmental changes (*47*, *48*). To gain further insight into the environmental changes that occur in the ER upon PA buildup, cells were fed 120 μM azido PA. The azide peak appears at 2196 cm^−1^ in the cell silent region and is expected to redshift upon exposure to a nonpolar environment. However, no 1734-cm^−1^ lipid peak was found in any cells fed azido PA, and the azide signal was only observed in LDs (fig. S14). This suggests that azido PA TAG precursors do not build up and form a lamellar gel state in the ER like PA-d_31_ and unlabeled PA. Unlike deuteration, which preserves the PA structure, incorporation of an azide slightly modifies the PA structure. Azido PA has a bulky azide group on the end of the hydrocarbon tail, which increases the area per molecule, reducing lipid thickness and restricting how closely molecules can pack together. Thus, our measurements suggest that azido PA has a melting point below PA and below physiological, 37°C. Azide FAs have widely been used in vibrational studies, yet, to our knowledge, the 1734-cm^−1^ shoulder has never been reported (*49*, *50*). This presents a pitfall in the use of azido FAs as the azide modification affects FA metabolism. In addition, this observation confirms the correlation between the lamellar gel state and DAG accumulation.

To further investigate the extent of this effect, 120 μM of a 1:10 mixture of azido PA to unlabeled PA was fed to cells and imaged 24 hours after feeding. Similarly, no 1734-cm^−1^ lipid peak was found in any cells. This suggests that even low concentrations of azido PA disrupt lamellar gel state formation. It is well established that OA prevents PA-induced apoptosis. Ratios of OA to PA as low as 1:25 have been recorded to have a protective effect against PA-induced apoptosis (*13*, *16*, *51*). Unsaturated long-chain FAs have lower melting temperatures and exist as liquids at room temperature. We hypothesize that the double bond of OA, similarly to the relatively bulky azide group of azido PA, disrupts formation of the lamellar gel state in the ER, resulting in unrestricted movement in the ER, maintaining metabolic flux, and sequestering FAs into LDs to prevent apoptosis.

As reported above, a few Huh-7 cells fed OA-d_33_ exhibited shifts in their ester carbonyl consistent with buildup of metabolic intermediates. To investigate whether this was correlated with a change in the state of the ER, 60 μM OA-d_33_ was fed to Huh-7 cells that were monitored by OPTIR approximately every 3 hours for 70 hours. As anticipated, most of the cells fed OA-d_33_ do not exhibit any lipid carbonyl shoulders or shifts. However, as previously observed, a few cells do exhibit broadening and shifts of lipid carbonyl peaks and corresponding CD_2_ shifts, indicating that there is a buildup of metabolites and a phase change near LD embedded in the ER (fig. S15, A to C). Still, this shift was found less frequently in OA-fed cells compared to PA-fed cells. When considering averaged spectra of all cells in each time point, there is no trend of 1734-cm^−1^ buildup (fig. S16A). Nor is there a consistent trend in the averaged second derivative spectra (fig. S16B). The spread of 1734-cm^−1^ intensity of all spectra per time point also does not have an obvious increasing and decreasing trend that is seen in PA-d_31_–fed cells (fig. S16C).

To quantify this, all spectra in the OA- and PA-fed time-lapse experiment where cells were fed 60 μM PA or OA conjugated to BSA at a 2:1 ratio and imaged every 3 hours were analyzed. In total, each time-lapse experiment included more than 100 cells, 400 LDs, and 1500 spectra (tables S2 and S3). A spectrum was considered to have a lipid carbonyl shift if there was a shoulder at 1738 cm^−1^ or below, after a background subtraction by shifting the baseline toward zero by subtracting a constant value of the intensity at 1816 cm^−1^ and normalizing the spectrum to 1744 cm^−1^. Between the time points of 1 to 70 hours, 40% of PA-fed cells and 23% of PA-fed LDs exhibited a lipid carbonyl shift in at least one spectrum, compared to 14% of OA-fed cells and 5% of OA-fed LDs (tables S2 and S3). Of the cells that exhibited the lipid carbonyl shift, 50% of PA-fed cell spectra had shifts, compared to 24% of OA-fed cell spectra. In general, hepatic LDs contain more TAGs than CEs (*52*, *53*). One possibility is that CEs can also undergo a phase transition, and cholesteryl oleate and cholesteryl palmitate have melting temperatures above physiological, ~44° and ~76°C, respectively (*54*, *55*). Thus, it stands that C─D shifts and DAG lipid carbonyl peaks are more common in PA-fed cells, as both palmitate TAG precursors and cholesteryl palmitate have higher than physiological melting temperatures. Only cholesteryl oleate has a higher than physiological melting temperature in OA-fed cells, resulting in less common C─D shifts. This suggests that not only are TAG precursors accumulating and forming a lamellar gel phase, but CEs contribute to this as well.

## DISCUSSION

Despite incredible advancements in understanding the roles LDs and PA play in metabolic disorders, many techniques cannot sufficiently answer the outstanding questions in the field. Lipidomics and biochemical assays have provided insight to how PA changes the FA profile but are lacking in spatial information essential to connect these effects to the cell at a subcellular level (*13*, *26*). Enzyme inhibition studies have spurred hypotheses on how PA disrupts metabolism but look only at a particular metabolic step rather than the entire cellular system or trigger off-pathway compensations (*17*). Optical imaging is a promising approach to spatially resolve cellular metabolism. Techniques including magnetic resonance imaging and fluorescence imaging have made great progress in visualizing cellular metabolism and have contributed to the development of drugs and disease prognosis (*56*). However, these techniques have their own limitations, whether it be sensitivity, suitability for dynamic imaging, or use of bulky and perturbative probes.

Vibrational microspectroscopy coupled with vibrational probes has the potential to be an extremely powerful technique in the field of cell biology. Vibrational probes are small and biorthogonal, allowing for their incorporation and detection among other exogenous cellular molecules (*49*). In addition, vibrational probes are environmentally sensitive providing information on the physical properties of the cell, which are difficult to obtain with conventional probes. As FAs are simple to label and easy for cells to uptake, many vibrational studies have monitored FA metabolism. Raman microspectroscopy has analyzed the deuterated and alkyne-tagged PA’s effect on cells and whole organisms (*37*, *57*, *58*). Tan *et al.* (*59*) found similar rates of FA uptake with 20 to 100 μM PA-d_31_, suggesting that membrane fluidity is similar across a broad range of feeding conditions. Confirming our observations, Fu *et al.* (*60*) observed strong C─D signals in the cytosol of PA-d_31_–fed cells but not OA-d_34_ fed cells. They hypothesized that phenotypic changes with PA-d_31_ feeding may be due to alterations in membrane structure or fluidity that are associated with cytotoxicity (*60*). Shen *et al.* (*61*) used the Raman C─D vibrational mode along with fluorescent stains and high-performance liquid chromatography–mass spectrometry to monitor the formation of solid-like domain separation in the ER induced by PA-d_31_ feeding in HeLa cells. However, like other Raman studies, they focus on the acyl chains, as C─H bonds are particularly Raman active while carbonyl bonds are weak (*62*). This presents a disadvantage of Raman imaging, as it is not possible to directly observe the chemistry that occurs at the lipid headgroup during TAG synthesis.

IR is more sensitive to carbonyl bonds, and, with the recent development of submicron OPTIR, IR imaging now provides sufficient resolution to probe carbonyl metabolism at the subcellular level. Recent OPTIR studies used labeled PA but, like previous Raman studies, focus on the C─H and C─D regions. Park *et al.* (*21*) monitored neutral lipid synthesis in live cells using PA-d_31_ but did not report any spectral shifts in the ester carbonyl region of TAGs/CEs. Shifts in the C─D region were attributed to heterogeneity of TAG acyl groups. While this could partially contribute to the broadening and shifts that we observe, the Fermi resonance in our data is strong spectral evidence of a liquid crystalline to lamellar gel phase change. Teng *et al.* (*63*) report FA desaturation of PA-d_31_ through monitoring the vibration of the unsaturated C─D bond. They do not report C─D shifts, and we do not observe an unsaturated C─D bond vibration upon PA-d_31_ feeding, but this may be due to a difference in cell lines. Bai *et al.* (*50*) also monitored lipid synthesis over various model systems but used azido-PA, which we found does affect FA metabolism by disrupting the formation of a lamellar gel state. While these studies advanced the field, our work highlights the importance of using probes that do not alter FA metabolism and that can directly monitor where the chemistry occurs, at the headgroup of the lipid.

DAGs are involved in many cellular functions, mostly as signaling molecules and precursors to glycerolipids (*64*). As a signaling molecule, it is thought that DAGs block insulin signaling, resulting in insulin resistance, a symptom of type 2 diabetes and hepatic steatosis, both of which are often simultaneously observed in patients (*65*, *66*). Thus, buildup of DAGs has been implicated in hepatic diseases, but the extent of its impact on PA lipotoxicity is still debated. Our evidence suggests that DAGs are a necessary component to PA lipotoxicity, as it is the longest lived lipid intermediate in the G3P pathway (Fig. 3). Previous work has implicated diacylglycerol transferase 1 (DGAT1), which converts DAGs to TAGs, as a reason for DAG buildup and inhibition of TAG synthesis (*17*, *27*). To test this, we treated Huh-7 cells fed 60 or 250 μM PA-d_31_ with 30 μM DGAT1 and 30 μM DGAT2 inhibitors (fig. S17, A to C). However, DAG buildup was not visible via OPTIR, indicating that Huh-7 cells have compensatory pathways that can be activated under these conditions (please see the Supplementary Materials for further discussion) (*26*, *67*, *68*). Unsaturated FAs are thought to be superior substrates for TAG synthesis, while saturated FAs and DAGs are thought to be poor substrates that impair cellular flux, leading to accumulation of saturated lipid species (*5*, *27*). This aligns well with other findings that have found that PA is less likely to be esterified into LD compared to OA, most likely due to TAG inhibition from saturated substrates (*12*, *13*, *27*). While our measurements agree that there are spatial differences in the esterification of OA and PA into LDs, we find that they result in a comparative buildup of TAG intermediates (Fig. 5C). Further, we also see that OA and PA have similar effects to cell viability at 60 μM. It is not until later time points and higher concentrations that cell viability greatly differs, with PA causing more cell death than OA (fig. S18, A to C). This confirms that there may be similar effects occurring in the cell, but once the buildup of TAG intermediates becomes overloading, the differences between OA and PA become more drastic. We also find that both OA and PA have similar upstream effects on metabolic pathways. Consistent with our past OA studies, compared to control conditions, cells fed ^13^C glucose and PA-d_31_ exhibit lower incorporation of ^13^C in TAGs/CEs (fig. S19) which we attribute to down-regulation of DNL-associated enzymes (*11*). Rather than saturated substrates inhibiting TAG enzymes in the G3P pathway, our measurements point toward an alternate, physical mechanism as the origin of DAG buildup in the ER.

In the ER, the C─D stretches of PA-derived intermediates were consistent with a lamellar gel state. As saturated lipids can pack tighter together than unsaturated lipids, increased membrane order induces ER stress (*26*, *69*). Many studies have linked PA to increased ER stress and, in turn, poor cellular health (*17*, *26*, *70*). This is due to altered lipid composition of the ER, which disrupts homeostasis and ultimately activates stress responses (*69*, *71*). Normally, to combat this, TAG synthesis enzymes relocalize from the ER to the budded LD surface via membrane tethers (*72*). However, upon elevated DAG levels, LD remains embedded in the ER. The conical shape of DAGs has a negative intrinsic curvature, which disfavors budding and promotes an embedded state of LDs (*4*, *73*). Our findings support this model. DAGs were only seen at the edges of LDs colocalized with the ER, suggesting that these LDs were still embedded in the ER. Further, the oval-shaped LDs that were observed imply that there are issues with proper budding. These were also the LDs that were more likely to accumulate DAGs. Furthermore, as azido PA cannot be packed as tightly as PA, it did not induce DAG accumulation. Similar effects are seen upon OA feeding. Numerous studies have reported that as little as 1:25, OA can reverse negative effects of PA (*13*, *14*, *51*). OA likely disrupts the packing of PA, thereby reducing ER stress due to its lower melting temperature and increased enzyme diffusion. Thus, it is a physical change rather than direct enzyme inhibition that gives rise to the altered PA metabolism. Although there are still many questions about LD growth and function, our findings contribute a spatial understanding of the role PA chemistry and disaturated DAGs play in LD dynamics and morphology.

Using OPTIR, we have visualized DAG accumulation upon PA feeding through the emergence of a time-dependent ester carbonyl peak and monitored physical properties of the ER through C─D stretches in the acyl chains. Notably, this chemistry could be tracked entirely label free. In addition, OPTIR allowed us to monitor lamellar gel and liquid crystalline lipid states in the ER and LDs using shifts in the C─D region. Through these observations, we have established a mechanism by which PA induces lipotoxicity in the cell. As saturated PAs are imported to the ER for TAG synthesis, acyl chain packing of PA and associated intermediates induces a phase change to an ordered lamellar gel state that slows diffusion of enzymes necessary for TAG synthesis. ER stress occurs through the accumulation of disaturated DAGs and other lipid intermediates as well as LD embedding in the ER. LD-organelle contact sites are disrupted as the LDs have not properly budded, interfering with lipid metabolism. This provides insight to how PA induces lipotoxicity and establishes OPTIR as a highly effective technique to monitor lipid metabolism.

## MATERIALS AND METHODS

### Materials

Unless otherwise specified, all materials were sourced from Sigma-Aldrich.

### FA conjugation

FA conjugation was performed as previously described using a protocol adapted by Shi *et al.* (*11*, *49*). Briefly, in a 2-ml clear glass container (VWR, Radnor, PA), PA-d_31_ (DLM 215, Cambridge Isotope Labs, Tewksbury, MA) or azido PA (CCT 1346, Vector Laboratories, Newark, CT) was dissolved in 29 mM NaOH and heated in a 70°C water bath until clear. The FA solution was then diluted to a total concentration of 3 mM with FA-free BSA (150 mg/ml; MP Biomedicals, Santa Ana, CA) dissolved in DPBS (Thermo Fisher Scientific, Waltham, MA). The solution was heated again at 37°C in a water bath until clear and filtered with a 0.2-μm sterile filter. Final ratios of PA to BSA were 2:1, in line with physiological ratios (*19*).

### Huh-7 cell culture

Huh-7 cells (a gift from the Yale Liver Center) were cultured as previously described (*11*). Briefly, cells were grown to 80% confluence in Dulbecco’s modified Eagle’s medium (DMEM) containing glucose (4.5 g/liter) and l-glutamine (Corning, Corning, NY) supplemented with 10% fetal bovine serum (FBS) and 1% penicillin/streptomycin (Thermo Fisher Scientific) under standard conditions (37°C, humidified atmosphere, 5% CO_2_). At 80% confluence, cells were trypsinized and replated on CaF_2_ coverslips (20 mm by 20 mm by 0.35 mm, Crystan, Poole, UK) in 35-mm sterile petri dishes (Corning). Cells were allowed to adhere to the coverslips overnight before FA feeding.

### Labeled PA cell feeding

Media was replaced with DMEM containing glucose (4.5 g/liter) and l-glutamine supplemented with 1% FBS, 1% penicillin/streptomycin, and 2 to 4% of unlabeled or labeled PA conjugated to BSA (final concentrations of PA between 60 and 120 μM). Media was not replenished with additional PA at any time point. Throughout the duration of the study, PA feeding was performed with 56 biological replicates.

### Cell viability assay

Cell viability assays were performed using the Cell Counting Kit-8 assay. Briefly, Huh-7 cells were seeded in 96-well plates at a density of 5000 cells per well in 100 μl of DMEM containing glucose (4.5 g/liter) and l-glutamine supplemented with 10% FBS and 1% penicillin/streptomycin. Cells were cultured in an incubator under standard conditions for 24 hours. Media was then removed and replaced with 100 μl of DMEM containing glucose (4.5 g/liter) and l-glutamine supplemented with 1% FBS, 1% penicillin/streptomycin, and the intended concentration of unlabeled or PA-d_31_ conjugated to BSA or OA-d_33_ conjugated to BSA. Cells were then incubated for the appropriate length of time, either 24, 48, or 72 hours. At each time point, 10 μl of Cell Counting Kit-8 solution was added to each well, the plate was incubated for 3 hours, and the absorbance at 450 nm was measured. Assay was performed with two biological replicates and three technical replicates.

### Live-cell imaging preparation

At the appropriate times after feeding, cells were rinsed twice with 3 ml of phosphate-buffered saline (PBS; Corning) and mounted in PBS on a glass microscopy slide (VWR) with a 10-μm double-sided tape spacer (Nitto, San Diego, CA).

### Fluorescence staining

Twenty-four hours after PA feeding, live cells were stained using an ER-ID Red assay kit (GRP CERTIFIED) (Enzo Life Sciences, Farmingdale, NY) and LipiSpot 488 Lipid Droplet Stain (Biotium, Fremont, CA) according to the manufacturers’ guides. Briefly, a staining solution containing 200 μl of 1× assay buffer provided by the ER-ID Red assay kit, 0.15 μl of ER-Red Detection Reagent, 0.15 μl of Hoechst 33342 Nuclear Stain, and 0.2 μl of LipiSpot 488 Lipid Droplet Stain was prepared. Cells were rinsed twice with PBS, followed by dispensing the 200 μl of prepared staining solution on the coverslip. Samples were incubated for 30 min at 37°C protected from light, after which samples were gently rinsed with 200 μl of 1× assay buffer and mounted on a glass microscopy slide as described above. Experiment was performed with 17 biological replicates.

### OPTIR data collection

All imaging was performed as previously described (*11*) on a mIRage-LS IR microscope (Photothermal Spectroscopy Corporation, Santa Barbara, CA) integrated with a four-module-pulsed quantum cascade laser system (Daylight Solutions, San Diego, CA) with a tunable range from 932 to 2348 cm^−1^. Bright-field optical images were collected using a low magnification ×10 refractive objective with a working distance of 15 mm. Spectra and IR images were collected in copropagating mode using a 40× Schwarzchild objective with a working distance of 8 mm in transmission mode. Data were collected with an IR laser power of 20% and a probe power in the range of 11%. All spectra and images were collected using PTIR Studio 4.6. (Photothermal Spectroscopy Corporation). For hyperspectral images, a step size of 250 nm and one acquisition was used. Spectra were collected using a high signal-to-noise ratio configuration of 200 cm^−1^/s. Spectra acquisition time was ~30 s per spectrum. For hyperspectral images, acquisition time was ~60 min. After hyperspectral imaging, LDs were checked using bright-field, or single wave number images ensure the cell did not move. If LDs moved during imaging, then the data were discarded.

### Fluorescence data collection

Fluorescent images were collected on the mIRage-LS IR microscope using a Prime BSI Express sCMOS (Teledyne, Thousand Oaks, CA). Fluorescent cells were excited using a Sola Light Engine (Lumencor, Beaverton, OR) and a green fluorescent protein (GFP) filter cube (TLV-U-FF-GFP, Thorlabs, Newton, NJ), a BFP filter cube (TLV-U-FF-BFP, Thorlabs), or a MCHE filter cube (TLV-U-FF-MCHC, Thorlabs). Images were collected using the 50× objective at 10% light-emitting diode intensity.

### Huh-7 transfection and staining

Huh-7 cells were transfected with ER-mEGFP. ER-mEGFP was a gift from S. Chiantia (Addgene plasmid #182876; http://n2t.net/addgene:182876; RRID:Addgene_182876) (*74*). Briefly, Huh-7 cells were cultured as described above. At 80% confluence, cells were trypsinized and replated in 35-mm glass-bottom petri dishes (Corning) in DMEM containing glucose (4.5 g/liter) and l-glutamine supplemented with 10% FBS only. To these cells, a plasmid-Lipofectamine 3000 (Invitrogen, Waltham, MA) complex at a 1:1 ratio (22.5 μl of Lipofectamine 3000 and 3 μg of ER-mEGFP plasmid) prepared in OptiMEM media (Gibco) was added. The cells were incubated at 37°C for 6 hours, when the media was replaced with DMEM containing glucose (4.5 g/liter) and l-glutamine supplemented with 10% FBS and 1% penicillin/streptomycin. After 24 hours, media was replaced with DMEM containing glucose (4.5 g/liter) and l-glutamine supplemented with 1% FBS, 1% penicillin/streptomycin, and 4% of PA conjugated to BSA or BSA (control). After another 24 hours, cells were fluorescently stained for the ER using the ER-ID assay kit as described above and imaged in FluoroBrite DMEM (Gibco).

### FRAP measurements

FRAP measurements were performed on a Zeiss LSM 880 confocal microscope. FRAP measurements of ER-mEGFP were performed by monitoring the fluorescence of the GFP with 488-nm excitation and 530.5-nm emission. Localization to the ER was confirmed by monitoring the fluorescence of the ER stain with 561-nm excitation and 628.5-nm emission. FRAP measurements were taken in the ER of control and cells fed PA-d_31_. Initial measurements of the fluorescence intensity were collected with defined regions of interest of 7.26 μm by 7.26 μm for five frames. The GFP was bleached using 488-nm light at a laser power of 100% with a bleach iteration of 15 for a total time of ~2 s. Recovery was monitored for 190 s at a scan speed of 1.9 frames per second.

FRAP curves were normalized to the prebleach intensity and baseline-corrected by setting the intensity of the first point after bleaching to 0. Curves were then fit to an exponential to determine diffusion time. FRAP experiments were performed with 19 biological replicates and 1 technical replicate.

### Data analysis

Fluorescence and bright-field images were processed in Fiji (National Institutes of Health, Bethesda, MD) (*75*). Spectra were analyzed in IGOR Pro 9 (Wavemetrics, Portland, OR). Live cell ratio images were generated in Python 3.10 in Colab (Google, Mountain View, CA) (*76*). Spectra were background corrected by shifting the baseline toward zero by subtracting by a constant value at 1816 cm^−1^ and then normalized to the 1744-cm^−1^ lipid carbonyl, unless otherwise specified.

### LD ellipticity

Ellipticity was calculated for LDs from cells 27 and 39 hours after being fed 60 μM PA-d_31_ or OA-d_33_ conjugated to BSA at a 2:1 ratio or control cells at 24 and 48 hours. Ellipticity of LDs was determined in Fiji by measuring the diameter across a LD *d*_1_ and dividing it by the diameter *d*_2_ where *d*_2_ is a 90° angle from *d*_1_. The diameter *d*_1_ was divided by *d*_2_ to obtain an ellipticity for the LD. Ellipticity was calculated for 25 LDs at 27 hours after feeding and 25 LDs at 39 hours after feeding for FA-fed cells and 25 LDs at 24 hours after feeding and 48 hours after feeding for control cells. Ellipticity was determined with 50 biological replicates and 1 technical replicate.

### Limit of detection

To determine the detection limit of the OPTIR, concentrations of 25, 15, 10, 5, 2, and 1 mM PA-d_31_ dissolved in ethanol and corresponding spectra were collected on the OPTIR.

#### Detection limit in C═O region

The carbonyl stretch of PA-d_31_ overlaps with the broad C─O stretch of ethanol. To resolve the PA-d_31_ carbonyl stretch, we approximate the C─O stretch of ethanol as linear over the frequency range of the PA-d_31_ carbonyl stretch and draw an artificial linear baseline below the PA-d_31_ carbonyl stretch (fig. S3, A and B). Briefly, the intensities of the raw spectra at two points at either end of the lipid carbonyl, 1744 and 1680 cm^−1^, were recorded and fit to a line. The resulting slope intercept formula is used to solve for the baseline intensity at 1708 cm^−1^ (*I*_B_). The baseline intensity at 1708 cm^−1^ was then subtracted from the intensity at 1708 cm^−1^ (*I*_S_) of the raw spectra to obtain a baseline-corrected intensity (*I*_C_). These values were then averaged over three spectra per concentration, plotted, and fit to a line to obtain the detection limit for the C═O region (fig. S3C).

#### Detection limit in C─D region

The *v*_as_ CD_2_ stretch of PA-d_31_ overlaps slightly with ethanol. To resolve differences in the *v*_as_ CD_2_ peaks, the baseline was shifted toward zero by subtracting a constant value of the intensity at 2164 cm^−1^ (bringing 2164 cm^−1^ to zero for each spectrum) and then normalized to the ethanol solvent band at 2258 cm^−1^ (fig. S3, D and E). The corrected intensities at 2200 cm^−1^ were then averaged over three spectra per PA-d_31_ concentration, plotted, and fit to a line to obtain the detection limit for the C─D region (fig. S3F).

### Hyperspectral image analysis

Hyperspectral images were used to determine spatial components of lipid peaks. The second derivative of hyperspectral images were calculated in IGOR Pro 9 to generate single wave number images composed of the second derivative of a specified wave number. The second derivative of the carbonyl region does not have any contribution from the amide I/water band, and the two lipid peaks at 1734 and 1744 cm^−1^ are separated and can be easily distinguished. Single wave number images depicting the intensity of the second derivative of 1744 and 1734 cm^−1^ were then generated using a code in Python 3.10 in Colab.

### Intermediate OPTIR spectra collection

The following intermediates were studied using OPTIR: tripalmitin, 1,2- dipalmitoyl-*sn*-glycerol, palmitoyl lysophosphatidic acid (Avanti Polar Lipids, Alabaster, AL), 1,2-dipalmitoyl-*sn*-glycero-3-phosphate (sodium salt) (Avanti Polar Lipids), and PA. Intermediates were dissolved at 25 mM in DCM (Thermo Fisher Scientific) and chloroform. Intermediate spectra were collected with an average of five technical replicates.

### Lipid extraction and TLC

Cells were cultured in flasks and treated with 60 μM BSA, OA-d_33_, or PA-d_31_. At 24 hours after treatment, cells were trypsinized and centrifuged at 3000*g* for 5 min. The supernatant was discarded, and cells were resuspended in PBS. This process was repeated a second time, where cells were resuspended in 0.8 ml of PBS. Lipids were isolated using the Bligh and Dyer method (*77*). Briefly, 3 ml of a cold chloroform/methanol (J.T. Baker, Phillipsburg, NJ) (1:2) solution was added to the resuspended cells, vortexed vigorously, and incubated on ice for 5 min. One milliliter of chloroform and 1 ml of PBS were added, and the solution was vortexed again followed by centrifuging at 1000*g* for 2 min. The bottom layer was recovered by a Pasteur pipette, and a second extraction was performed on the cells by adding 1 ml of chloroform and centrifuging again at 1000*g* for 2 min. The bottom layer was recovered again and dried under nitrogen, resuspended in 20 μl of chloroform, and spotted on a silica gel plate. Lipids were separated using a mobile phase adapted from Chung *et al.* (*78*) of hexane (Thermo Fisher Scientific):diethyl ether (Thermo Fisher Scientific):acetic acid (J.T. Baker) (80:20:1; v/v/v). TLC plates were then dried for 20 min and stained with a 0.2% solution of amido black (Thermo Fisher Scientific). The TLC plate was quantified in Fiji by selecting each lane using the rectangle tool and plotting each lane to visualize the density of each band as a peak and measuring that value. Lipid extraction and TLC experiments were performed with 21 biological replicates and 1 technical replicate.

### DGAT inhibition

Huh-7 cells were cultured as described above and trypsinized at 80% confluency. They were then plated on CaF_2_ coverslips in 35-mm sterile petri dishes and allowed to adhere to the coverslips overnight before FA feeding. Cells were then fed 60 or 250 μM PA-d_31_ and 30 μM DGAT1 inhibitor (T863, MedChemExpress, Princeton, NJ) and 30 μM DGAT2 inhibitor (PF06424439, MedChemExpress). Cells were incubated for 16 hours and then analyzed via OPTIR. Experiments were performed with an average of seven biological replicates and 1 technical replicate.

## Supplementary Materials

### This PDF file includes:

Supplementary Text Figs. S1 to S19 Tables S1 to S3 References
