# Supplementary material for: Optical photothermal infrared imaging of fatty acid esterification in the ER of living cells
Source: Sci Adv. 2026 Jul 8;12(28):eaed6477. doi: 10.1126/sciadv.aed6477 (PMC13344281; doi:10.1126/sciadv.aed6477)
Supplement: Supplementary file 1 — Supplementary Text Figs. S1 to S19 Tables S1 to S3 References [file sciadv.aed6477_sm.pdf]

Science Advances
AAAS

Supplementary Materials for

Optical photothermal infrared imaging of fatty acid esterification in the ER of living cells

Hannah B. Castillo and Caitlin M. Davis

Corresponding author: Caitlin M. Davis, c.davis@yale.edu

Sci. Adv. **12**, eaed6477 (2026)
DOI: 10.1126/sciadv.aed6477

This PDF file includes:

Supplementary Text
Figs. S1 to S19
Tables S1 to S3
References

Diacylglycerol Transferase (DGAT) 1&2 Inhibition

Huh-7 cells were fed increasing concentrations of deuterated palmitic acid (PA-d_{31}) and administered 30 μM of DGAT1 and 30 μM DGAT2 inhibitors. After 16 hours, in accordance with previous protocols (*26),* cells were imaged live via optical photothermal infrared (OPTIR) microspectroscopy. At 60 μM of PA-d_{31}, no C-D stretches were visible, nor any 1734 cm^{-1} shoulder (**Fig. S17A).** At 250 μM of PA-d_{31}, C-D stretches were visible, but no 1734 cm^{-1} shoulder was detectable (**Fig. S17B**). For comparison, 250 μM of PA-d_{31} was fed to cells without any DGAT inhibition and strong 1734 cm^{-1} signal was observed (**Fig. S17C**).

Previous studies show that the effect of DGAT1 and 2 inhibition is highly dependent on the type of fatty acid (FA) that cells are fed. Løvsletten et al. reported that lipid synthesis in DGAT1 and DGAT2 inhibited cells fed oleic acid (OA) is redirected towards phospholipids (*67*). Pérez-Martí et al. found that DGAT1 and 2 inhibited cells fed palmitic acid (PA) exhibit strong metabolic buildup of intermediates in the glycerol-3-phosphate (G3P) pathway, including lysophosphatidic acid (LPA), PA, and diacylglycerol (DAG) (*26).* But both Pérez-Martí et al. and Longo et al. showed that co-feeding of PA and OA reduced lipid content in DGAT inhibited cells while also reducing markers of endoplasmic reticulum (ER) stress and oxidative stress, implying that although triacylglycerol (TAG) synthesis is halted, DAGs are not accumulating in the ER or causing ER stress (*26, 68*). Pérez-Martí et al. hypothesized that monounsaturated FAs like OA already stored in lipid droplets (LDs) could be released to facilitate esterification of PA into LDs (*26).* Indeed, prefeeding OA to cells and then introducing DGAT inhibitors and PA reduced cytotoxicity. Unlike the induced renal epithelial cells (iRECS) that Pérez-Martí et al. used, the hepatocyte Huh-7 cell line we used is natively rich in LDs (*26).* Thus, our experiments feeding PA and treating with DGAT1 and DGAT2 inhibitors are consistent with the results of the pretreatment experiment by Pérez-Martí et al. This suggests that monounsaturated FAs stored in the LDs of Huh-7 cells are released during DGAT 1 and 2 inhibition and able to buffer the overload of saturated PA fed to cells thereby redirecting lipid synthesis and reducing cytotoxicity.

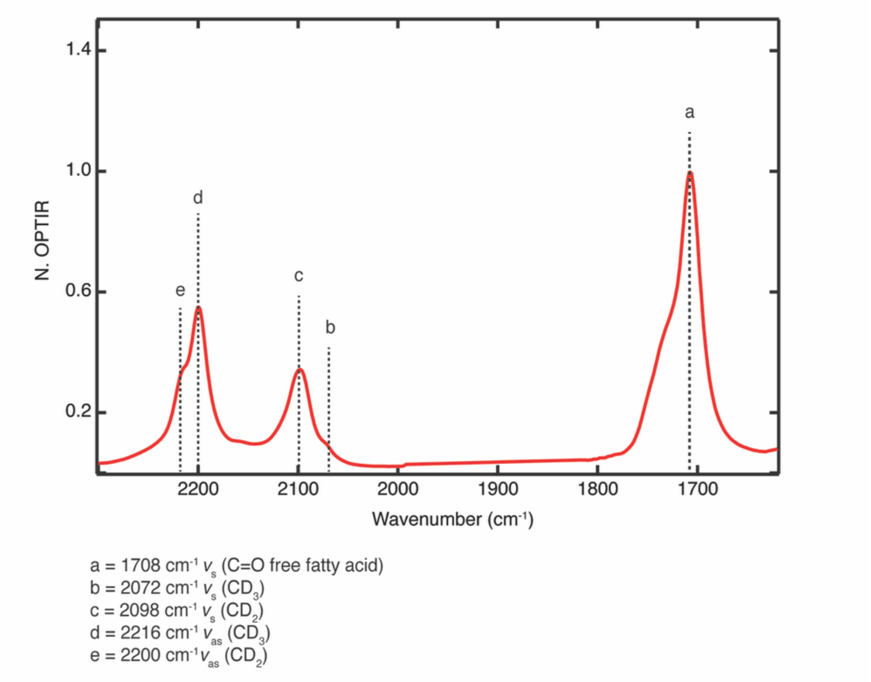

Fig. S1. Full assignments of the optical photothermal infrared (OPTIR) spectrum of 50 mM deuterated palmitic acid (PA-d_{31}) in chloroform ($CHCl_3$) (*79*, *80*). Figure displays one technical replicate.

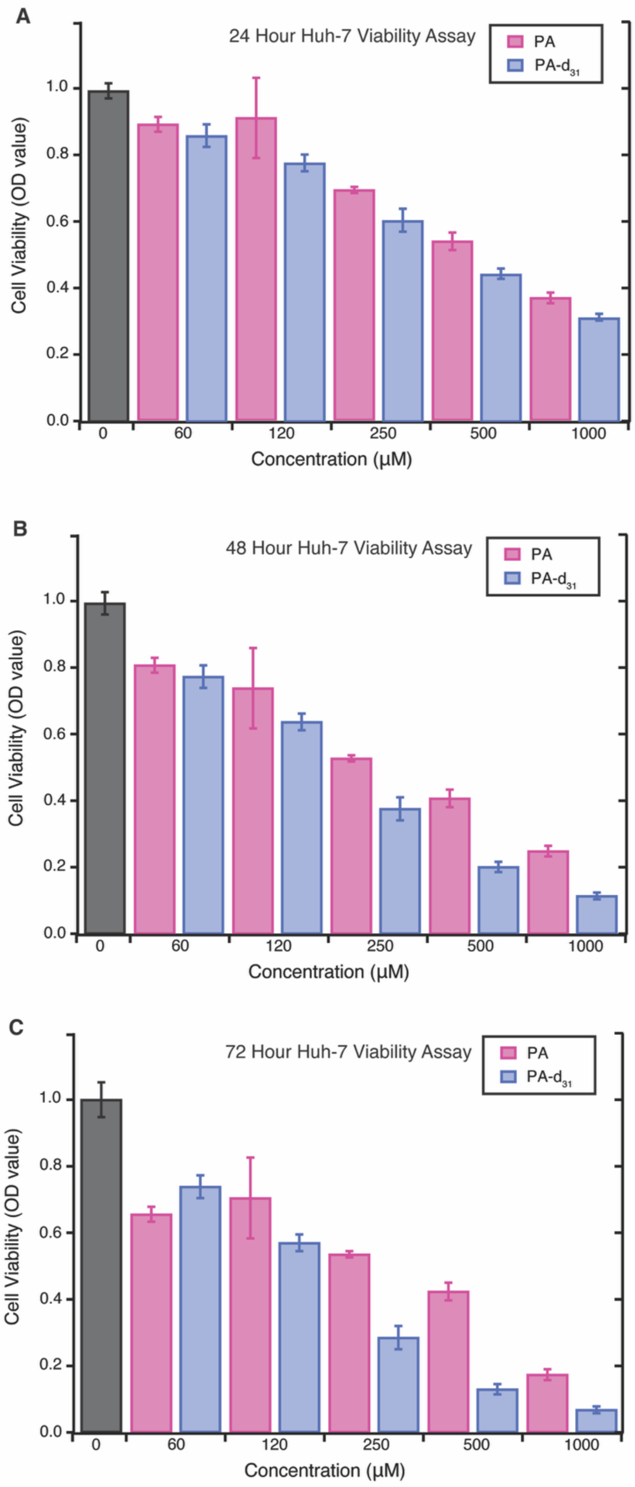

Fig. S2. Cell viability assays of cells fed unlabeled palmitic acid (PA) versus deuterated palmitic acid (PA-d$_{31}$) at concentrations of 60, 120, 250, 500, and 1000 µM PA conjugated to bovine serum albumin (BSA) at a 2:1 ratio. (A) Cell viability assay at 24 hours. **(B)** Cell viability assay at 48 hours. **(C)** Cell viability assay at 72 hours. Error bars are one standard deviation of the average cell viability. Figure displays two biological replicates and three technical replicates.

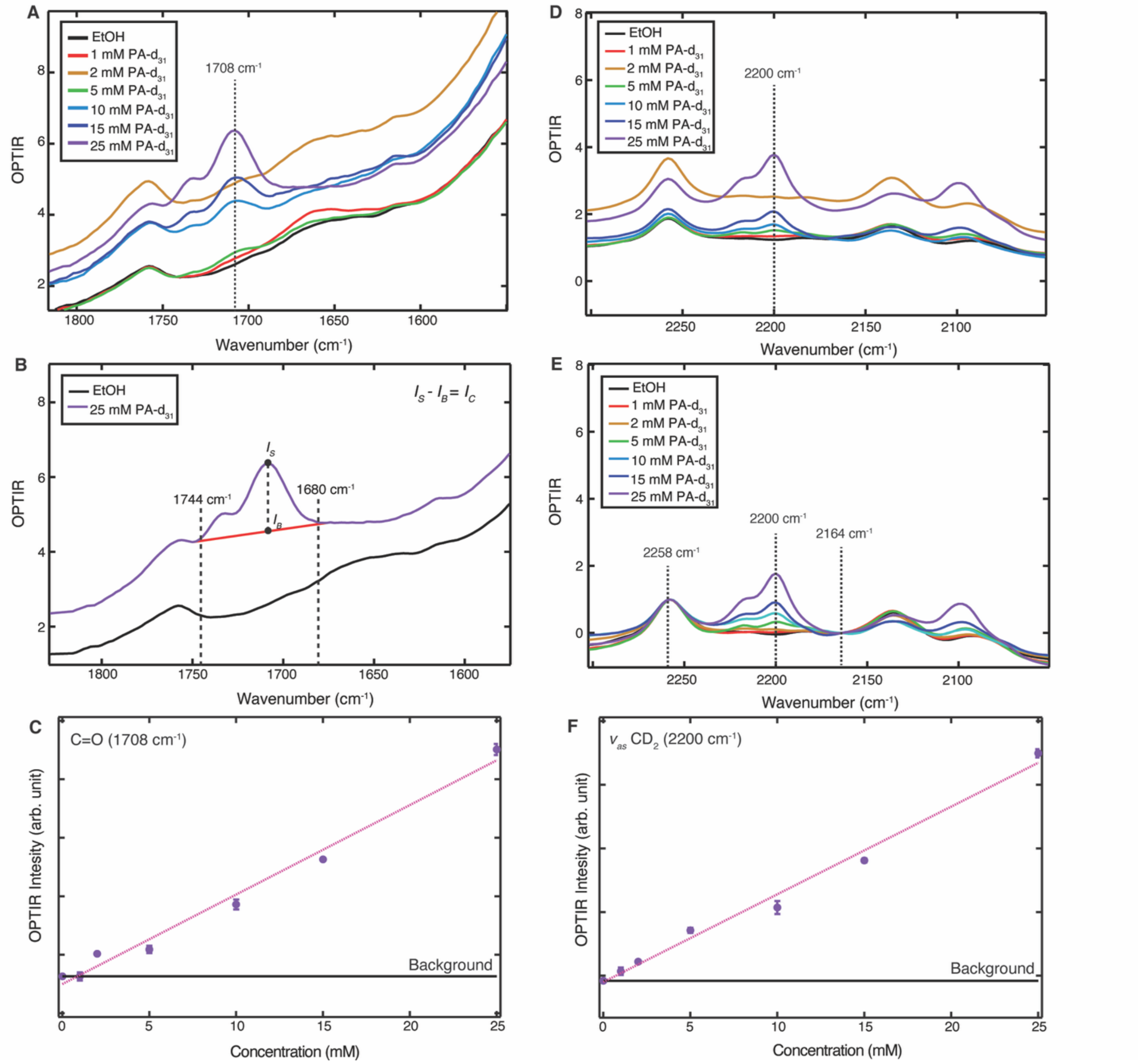

Fig. S3. Detection limit of the optical photothermal infrared (OPTIR) instrument. (A) Detection limit for the C=O stretch was determined by comparing spectra of 25 mM, 15 mM, 10 mM, 5 mM, 2 mM, and 1 mM of deuterated palmitic acid (PA-d_{31}) in ethanol (EtOH). **(B)** To resolve the C=O peaks, the C-O stretch of EtOH was approximated to be linear and an artificial linear baseline was drawn below the PA-d_{31} carbonyl stretch. The line was fit to determine the baseline intensity (I_B) and subtracted by the intensity of the raw spectra (I_S) to obtain the corrected intensity (I_C). **(C)** Detection limit for the C=O stretch was determined to be approximately 0.86 mM. **(D)** Detection limit for the v_{as} CD_2 stretch was determined by comparing spectra of 25 mM, 15 mM, 10 mM, 5 mM, 2 mM, and 1 mM of PA-d_{31} in EtOH. **(E)** To resolve the v_{as} CD_2 stretches, the baseline was shifted towards zero by subtracting a constant value of the intensity at 2164 cm^{-1} (bringing 2164 cm^{-1} to zero for each spectrum) and then normalized to the EtOH solvent band at 2258 cm^{-1}. **(F)** The detection limit for the v_{as} CD_2 carbonyl was determined to be approximately 0.14 mM.

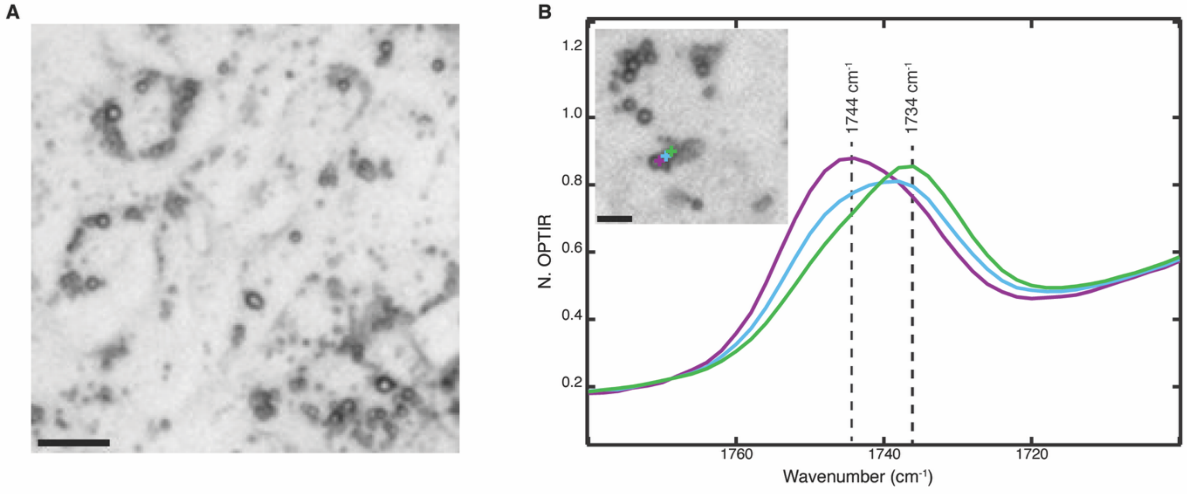

Fig. S4. Representative spectra collected in a lipid droplet (LD) of a live Huh-7 cell 24 hours after feeding 60 µM unlabeled palmitic acid (PA) conjugated to bovine serum albumin (BSA) at a 2:1 ratio. (A) Brightfield image of live Huh-7 cells 24 hours after feeding 60 µM of unlabeled PA conjugated to BSA at a 2:1 ratio. Scale bar is 20 µm. **(B)** Green, purple, and blue spectra collected 24 hours after feeding 60 µM of unlabeled PA conjugated to BSA at a 2:1 ratio. The presence of a shoulder at 1734 cm^{-1} with unlabeled PA feeding is consistent with deuterated palmitic acid (PA-d_{31}) feeding. Inset: Colored crosses indicate the location the corresponding colored spectra were collected in a LD of a live Huh-7 cell. Scale bar is 10 µm.

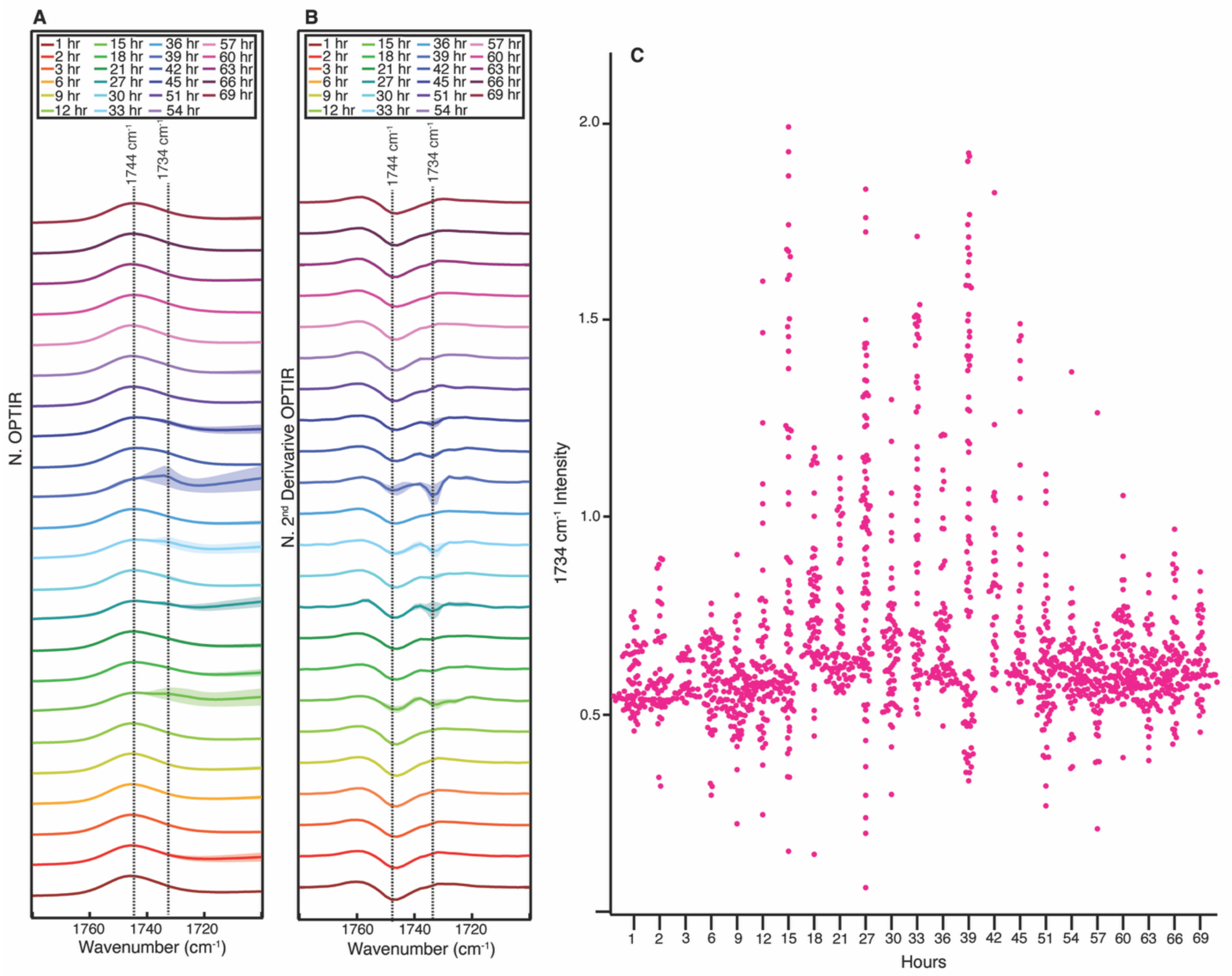

Fig. S5. Trend of 1734 cm^{-1} intensity throughout 70 hours (hr) of cells fed 60 µM of deuterated palmitic acid (PA-d_{31}). (**A**) Averaged spectra of all cells for each timepoint showing the increase and decrease of 1734 cm^{-1} intensity throughout 70 hr. Shading indicates standard error of the averaged spectra. (**B**) Averaged second derivative spectra of all cells for each timepoint showing the 1734 cm^{-1} trend. Shading indicates standard error of the averaged spectra. (**C**) Intensity at 1734 cm^{-1} of each spectrum averaged together in (A) after normalizing to 1744 cm^{-1} and background correcting by shifting the baseline towards 0 by subtracting a constant value of the intensity at 1816 cm^{-1}. The spread of 1734 cm^{-1} intensity throughout 70 hr confirms the trends in the averaged spectra. Each time point represents an average of six biological replicates and one technical replicate. Two technical replicates were required to capture all timepoints.

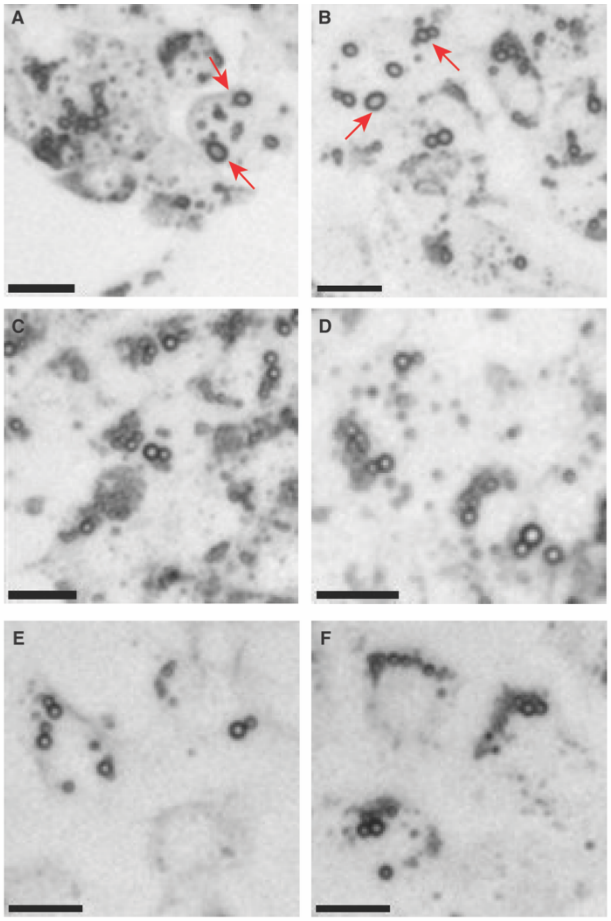

Fig. S6. Irregularly shaped lipid droplets (LDs) form when Huh-7 cells are fed palmitic acid (PA) but not oleic acid (OA) or bovine serum albumin (BSA). Representative abnormal LDs are apparent 39 hours **(A)** and 27 hours **(B)** after cells are fed 60 µM of deuterated palmitic acid (PA-d_{31}) conjugated to BSA at a 2:1 ratio. Red arrows point out several oval shaped LDs. Representative LDs in cells fed 60 µM of deuterated oleic acid (OA-d_{33}) conjugated to BSA at a 2:1 ratio are spherical at the same time points, **(C)** 39 hours and **(D)** 27 hours. Representative LDs in control cells fed BSA **(E)** and **(F)** are also regularly shaped. Scale bars are all 20 µm.

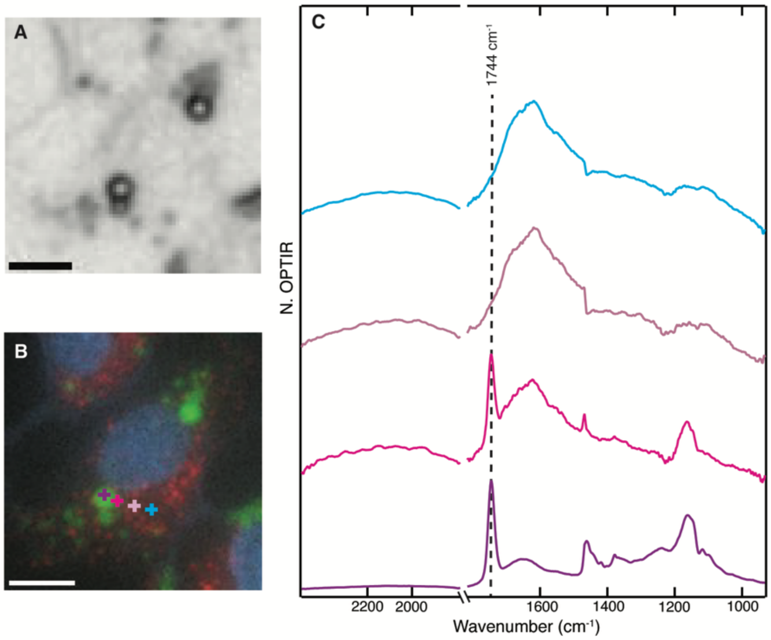

Fig. S7. Control cell spectra of lipid droplet (LD) and the endoplasmic reticulum (ER). (A) Brightfield image of a live Huh-7 control cell. **(B)** Fluorescence stains of ER (red), nucleus (blue), and LD (green) of the live Huh-7 control cell. Colored crosshairs indicate where corresponding colored spectra **(C)** were collected in a linescan across the LD and ER. Scale bars are 10 µm. **(C)** Spectra collected at the center of a LD, edge of a LD, and in the ER.

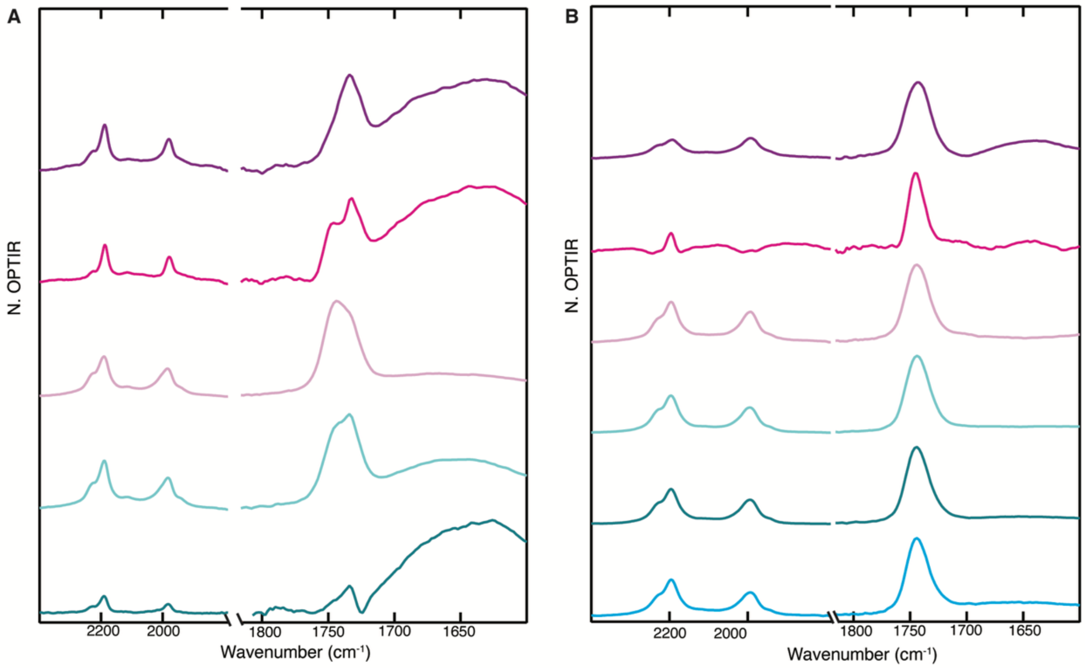

Fig. S8. Full spectra from linescans across lipid droplets (LDs) in Fig. 4. (A) Full spectra corresponding to the points in **Fig. 4G** where a linescan was collected across a LD near the endoplasmic reticulum (ER). **(B)** Full spectra corresponding to the points in **Fig. 4H** where a linescan was collected across a LD away from the ER.

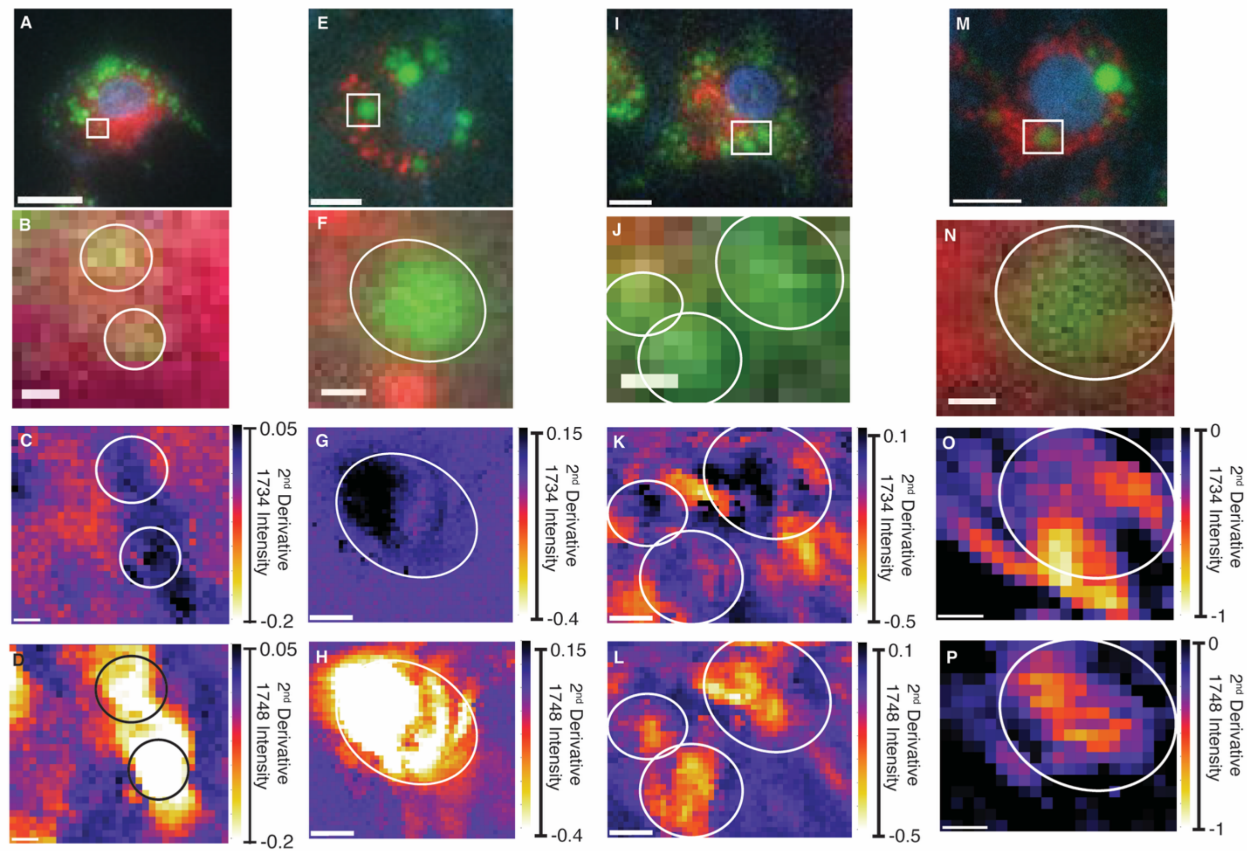

Fig. S9. Spatial trends of 1734 cm^{-1} intensity are confirmed in cells imaged 24 hours after feeding of 120 μM deuterated palmitic acid (PA-d_{31}) conjugated to bovine serum albumin (BSA) at a 2:1 ratio and stained for the endoplasmic reticulum (ER. red), lipid droplets (LDs, green), and nucleus (blue). (A) Fluorescent image of a Huh-7 cell with a white box indicating where hyperspectral imaging was performed. Scale bars are 20 μm. **(B)** Fluorescence signal in the region where the hyperspectral was collected. LDs are outlined in white. Scale bar is 1 μm. **(C)** A single wavenumber image depicting the intensity of the second derivative of 1734 cm^{-1}. LDs are outlined in white. Scale bar is 1 μm. **(D)** A single wavenumber image depicting the intensity of the second derivative of 1748 cm^{-1}. LDs are outlined in black. Scale bar is 1 μm. **(E)** Fluorescent image of a Huh-7 cell with a white box indicating where hyperspectral imaging was performed. Scale bars are 10 μm. **(F)** Fluorescence signal in the region where the hyperspectral was collected. LDs are outlined in white. Scale bar is 1 μm. **(G)** A single wavenumber image depicting the intensity of the second derivative of 1734 cm^{-1}. LDs are outlined in white. Scale bar is 1 μm. **(H)** A single wavenumber image depicting the intensity of the second derivative of 1748 cm^{-1}. LDs are outlined in white. Scale bar is 1 μm. **(I)** Fluorescent image of a Huh-7 cell with a white box indicating where hyperspectral imaging was performed. Scale bars are 10 μm. **(J)** Fluorescence signal in the region where the hyperspectral was collected. LDs are outlined in white. Scale bar is 2 μm. **(K)** A single wavenumber image depicting the intensity of the second derivative of 1734 cm^{-1}. LDs are outlined in white. Scale bar is 2 μm. **(L)** A single wavenumber image depicting the intensity of the second derivative of 1748 cm^{-1}. LDs are outlined in white. Scale bar is 2 μm. **(M)** Fluorescent image of a Huh-7 cell with a white box indicating where hyperspectral imaging was performed. Scale bars are 10 μm. **(N)** Fluorescence signal in the region where the hyperspectral was collected. LDs are outlined in white. Scale bar is 1 μm. **(O)** A single wavenumber image

depicting the intensity of the second derivative of 1734 cm^{-1}. LDs are outlined in white. Scale bar is 1 µm. **(P)** A single wavenumber image depicting the intensity of the second derivative of 1748 cm^{-1}. LDs are outlined in white. Scale bar is 1 µm.

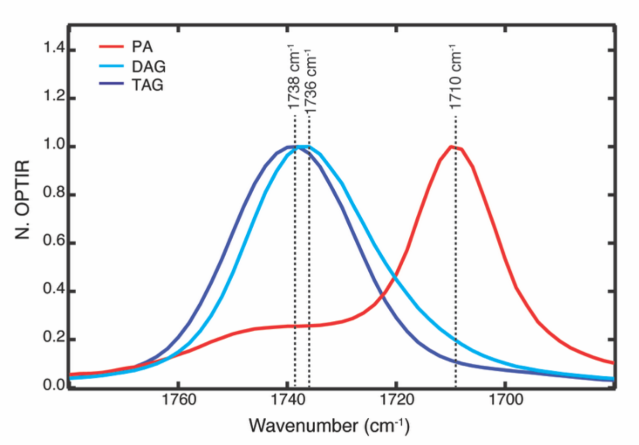

Fig. S10. Optical photothermal infrared (OPTIR) spectra of 25 mM triacylgyceride precursors in dichloromethane (DCM). Palmitic acid (PA, red), 1,2 dipalmitin (DAG, blue), and tripalmitin (TAG, navy). Figure displays one technical replicate.

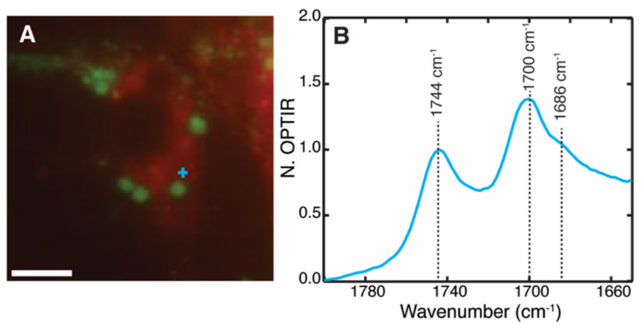

Fig. S11. Spectra from Huh-7 cells fed 120 µM of ^{13}C palmitic acid (PA) conjugated to bovine serum albumin (BSA) at a 2:1 ratio. (A) Fluorescence image of a live Huh-7 cell fed ^{13}C PA and stained for the endoplasmic reticulum (ER, red) and lipid droplets (LDs, green). Scale bar is 10 µm. (B) Optical photothermal infrared (OPTIR) spectrum at the blue cross hairs in (A) show both ^{13}C triacyclglycerols (TAGs) and diacylglycerol (DAGs) present in the ER.

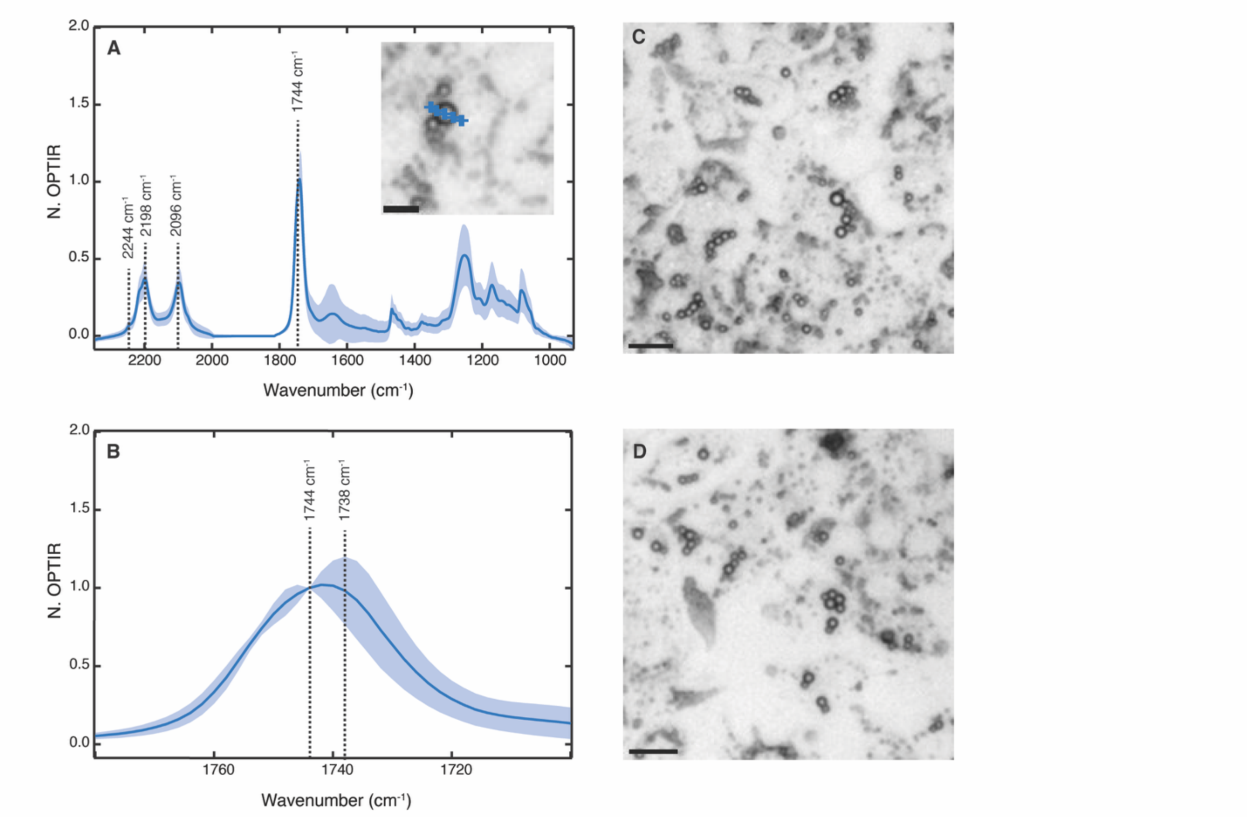

Fig. S12. Cells fed 60 μM of deuterated oleic acid (OA-d_{33}) conjugated to bovine serum albumin (BSA) at a 2:1 ratio exhibit lipid carbonyl broadening and shoulders. **(A)** Averaged normalized optical photothermal infrared (OPTIR) spectrum collected across a lipid droplet (LD) in a Huh-7 cell 40 hours after feeding 60 μM of OA-d_{33} (inset). The blue markers represent where spectra were collected. Scale bar is 10 μm. **(B)** A closeup of the lipid carbonyl region from 1700 cm^{-1} to 1780 cm^{-1}. Shading is one standard deviation of the average normalized spectra. (**C-D**) Representative brightfield images of cells 40 hours after feeding 60 μM of OA-d_{33}. Scale bars are 20 μm.

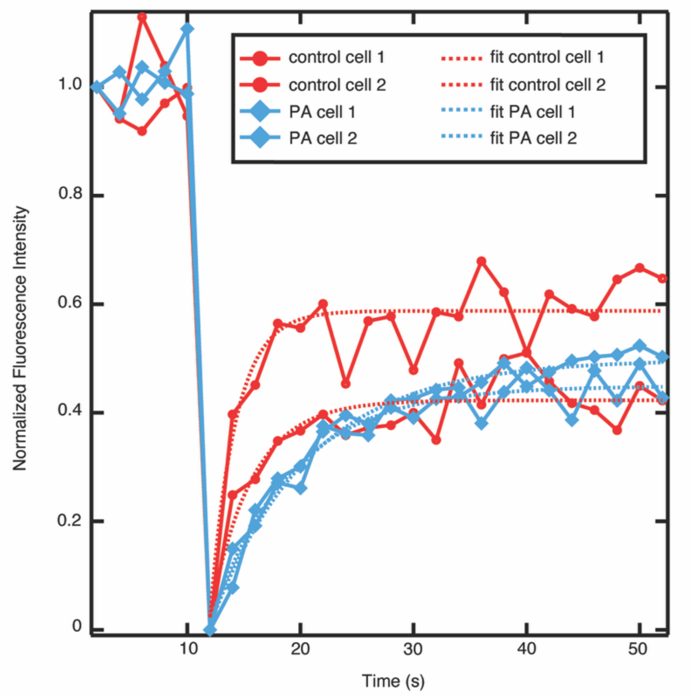

Fig. S13. Fluorescence recovery after photobleaching (FRAP) curves of control cells and cells fed 120 µM deuterated palmitic acid (PA-d_{31}) conjugated to bovine serum albumin (BSA) at a 2:1 ratio. Data fit to an exponential. Figure displays two biological replicates and one technical replicate.

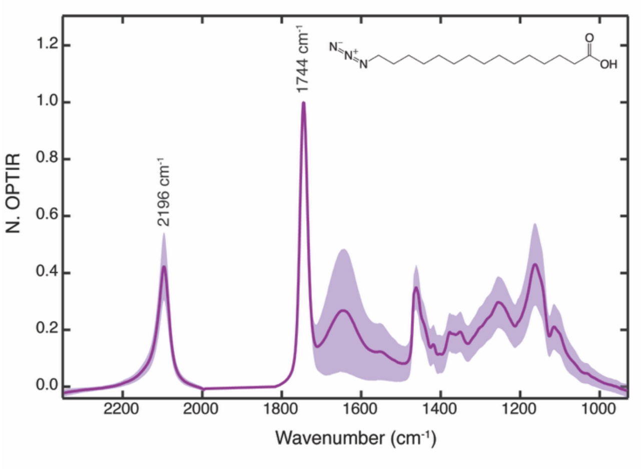

Fig. S14. Average normalized optical photothermal infrared (OPTIR) spectrum of a representative lipid droplet (LD) in a Huh-7 cell 24 hours after 120 µM of azido palmitic acid (PA) conjugated to bovine serum albumin (BSA) at a 2:1 ratio feeding. Shading is one standard deviation of the average normalized spectra.

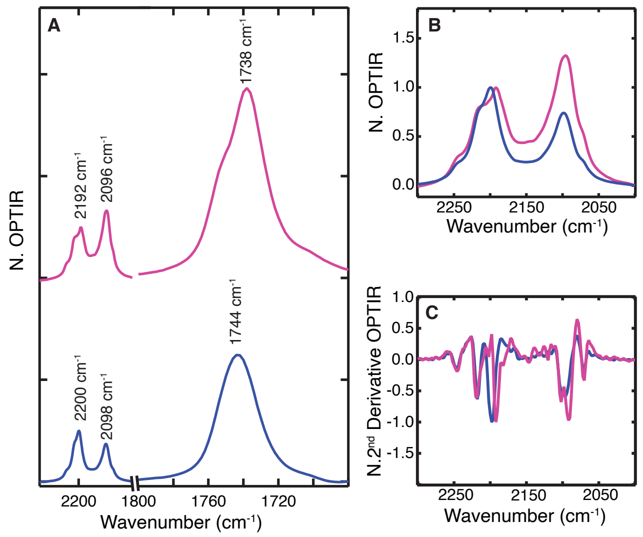

Fig. S15. Comparison of the C-D region of representative Huh-7 cells fed 60 μM deuterated oleic acid (OA-d_{33}) conjugated to bovine serum albumin (BSA) at a 2:1 ratio. (A) Representative spectra with (pink) and without (blue) strong 1738 cm^{-1} intensity. A redshift of the asymmetric and symmetric CD_2 stretches is correlated with the appearance of a 1738 cm^{-1} stretch alongside the 1744 cm^{-1} lipid carbonyl. Enlarged view of the C-D region and its corresponding second derivative spectra is shown in **(B)** and **(C)**, respectively. Spectra were normalized by prominent peak heights in the C-D region.

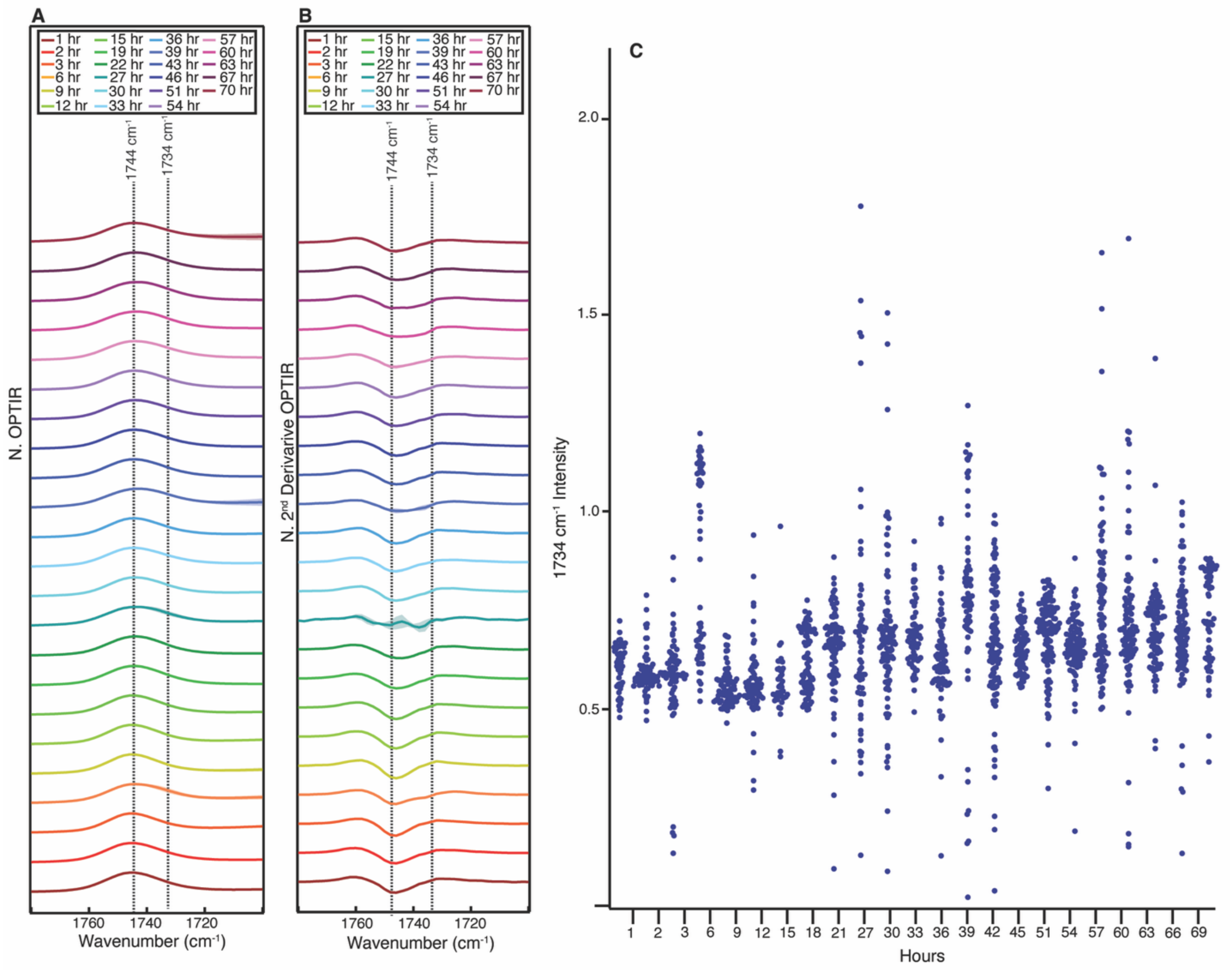

Fig. S16. Trend of 1734 cm^{-1} intensity throughout 70 hours (hr) for cells fed 60 µM of deuterated oleic acid (OA-d$_{33}$). (**A**) Averaged spectra of all cells for each timepoint showing no general increase in 1734 cm^{-1} shoulder throughout 70 hr. Shading indicates standard error of the averaged spectra. (**B**) Averaged second derivative spectra of all cells for each timepoint showing no significant 1734 cm^{-1} trend. Shading indicates standard error of the averaged spectra. (**C**) Intensity at 1734 cm^{-1} of each spectrum average together in (A) after normalizing to 1744 cm^{-1} and background correcting by shifting the baseline towards 0 by subtracting a constant value of the intensity at 1816 cm^{-1}. The spread of 1734 cm^{-1} intensity throughout 70 hr confirms the trends in the averaged spectra. Each time point represents an average of six biological replicates and one technical replicate. Two technical replicates were required to capture all timepoints.

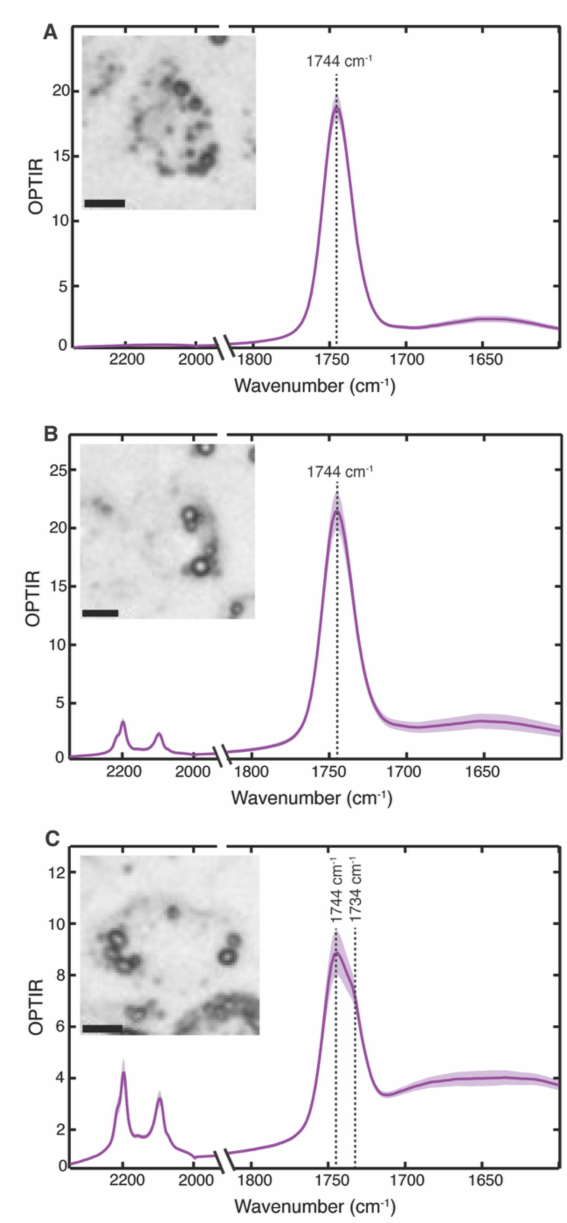

Fig. S17. Feeding diacylglycerol transferase 1/2 (DGAT1/2) inhibitors does not result in buildup of the 1734 cm^{-1} shoulder. (**A**) Averaged spectra from lipid droplets (LDs) of a Huh-7 cell 24 hours after being fed 60 μM deuterated palmitic acid (PA-d_{31}) and 30 μM DGAT1/2 inhibitors (inset). (**B**) Averaged spectra from LDs of a Huh-7 cell 16 hours after being fed 250 μM PA-d_{31} and 30 μM DGAT1/2 inhibitors (inset). (**C**) Averaged spectra from LDs of a Huh-7 cell 16 hours after being fed 250 μM PA-d_{31} (inset). All scale bars are 10 μm.

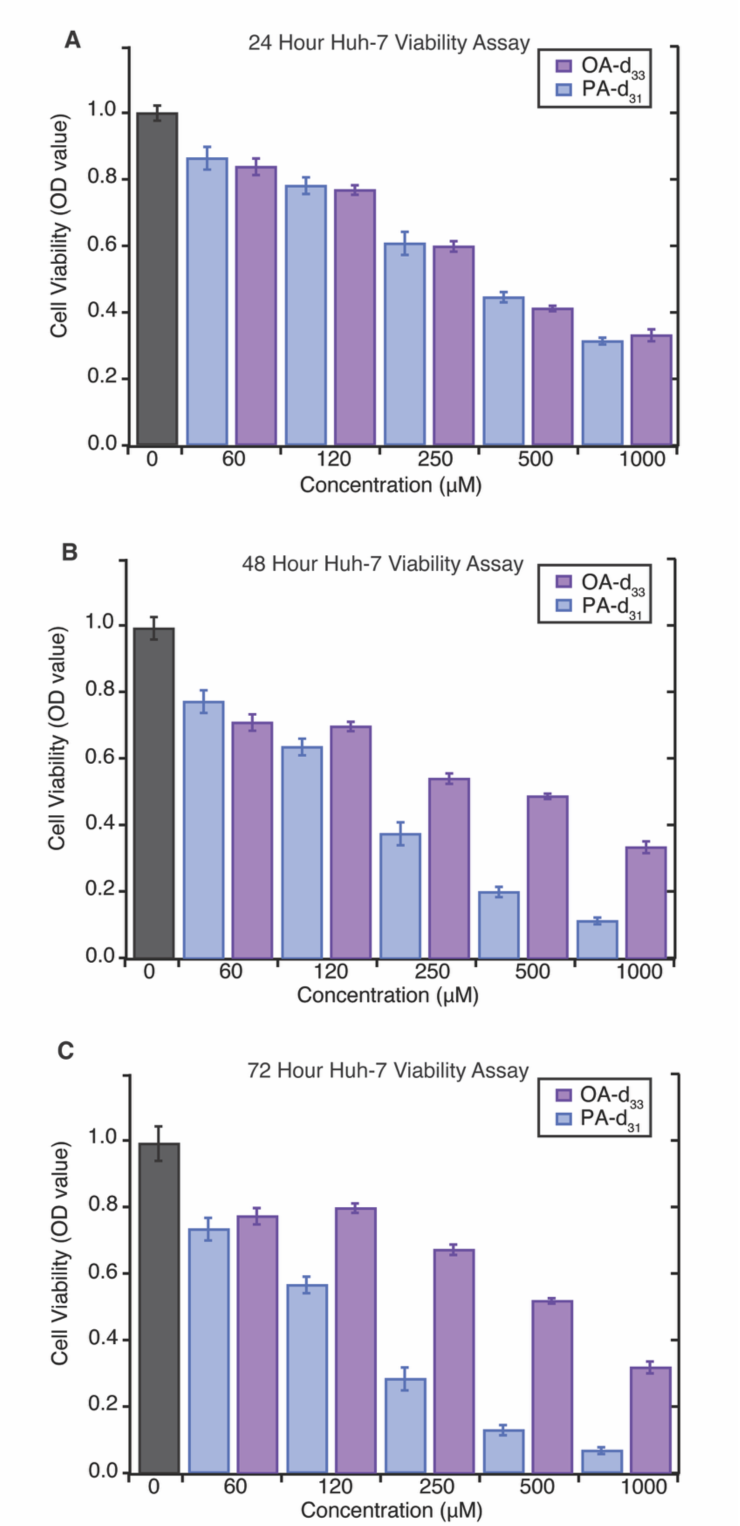

Fig. S18. Cell viability assays of cells fed deuterated oleic acid (OA-d_{33}) versus deuterated palmitic acid (PA-d_{31}) at concentrations of 60, 120, 250, 500, and 1000 µM conjugated to bovine serum albumin (BSA) at a 2:1 ratio. **(A)** Cell viability assay at 24 hours. **(B)** Cell viability assay at 48 hours. **(C)** Cell viability assay at 72 hours. Error bars are one standard deviation of the average cell viability. Figure displays two biological replicates and three technical replicates.

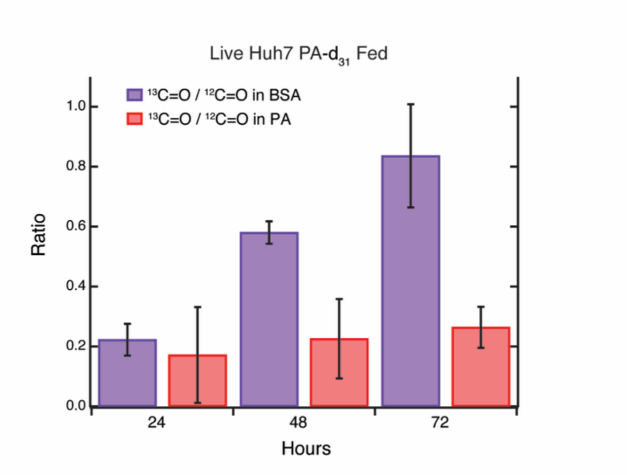

Fig. S19. Rates of *de novo* lipogenesis (DNL) decrease upon 60 µM deuterated palmitic acid (PA-d_{31}) conjugated to bovine serum albumin (BSA) at a 2:1 ratio feeding (red) compared to control cells fed BSA only (purple). Error bars represent one standard deviation of average of the averages of the cells at each time point including propagated error from the standard error of each cell. Figure displays 14 biological replicates and 1 technical replicate. PA, palmitic acid.

Table S1. Calculated ellipticity of lipid droplets (LDs) from cells fed 60 µM deuterated palmitic acid (PA-d_{31}) or deuterated oleic acid (OA-d_{33}) conjugated to bovine serum albumin (BSA) at a 2:1 ratio or control cells. Ellipticity was calculated across 50 LDs total from cells fed 60 µM PA-d_{31} or OA-d_{33} conjugated to BSA at a 2:1 ratio or control cells. Ellipticity for 25 LDs from the 27 hour timepoint and 25 LDs from the 39 hour timepoint were averaged for FA fed cells and 25 LDs from the 24 hour timepoint and 25 LDs from the 48 hour timepoint for control cells were averaged. Totals are displayed as averages and one standard deviation of the average.

**Hour**	**D_1/D_2 of PA-d_{31} fed cells**	**D_1/D_2 of OA-d_{33} fed cells**	**Hour**	**D_1/D_2 of control cells**
**27 Hours**	0.88	0.95	**24 Hours**	0.96
	0.76	0.96		0.97
	0.77	0.92		0.86
	0.74	0.95		1.00
	0.92	0.88		0.97
	0.97	0.98		0.97
	0.77	0.97		0.98
	0.98	0.91		0.97
	1.00	1.00		0.96
	0.84	0.78		0.97
	0.85	0.95		0.97
	0.66	1.00		0.97
	0.83	1.00		0.89
	0.79	0.96		0.87
	0.96	0.86		0.89
	0.71	1.00		0.94
	0.92	0.96		0.95
	0.89	0.94		0.85
	0.73	0.97		1.00
	0.98	0.94		0.81
	0.76	0.95		0.63
	0.72	0.95		0.98
	0.98	0.91		0.90
	0.89	0.96		0.94
**39 Hours**	0.75	0.98	**48 Hours**	0.84
	0.84	0.93		0.98
	0.74	0.93		1.00
	1.00	0.96		1.00
	0.71	0.95		0.93
	0.76	0.98		1.00
	0.73	1.00		0.98

	0.75	1.00		0.97
	0.72	1.00		0.99
	0.88	0.76		0.96
	0.85	0.94		0.94
	0.82	0.97		0.98
	0.73	0.97		0.95
	0.88	0.74		0.95
	0.62	0.91		0.86
	0.64	0.91		0.95
	0.71	1.00		0.94
	0.80	0.80		0.91
	0.88	0.98		0.97
	0.96	0.83		0.91
	0.80	0.86		0.95
	0.98	1.00		0.97
	0.98	0.94		0.98
	0.80	0.93		1.00
	0.66	0.98		1.00
	0.62	0.93		0.93
**Average**	**0.8 ± 0.1**	**0.93 ± 0.06**		**0.94 ± 0.6**

Table S2. Counts of 1734 cm^{-1} shoulder throughout 70 hours of deuterated palmitic acid (PA-d_{31}) feeding. Total numbers of Cells, lipid droplets (LDs), and spectra that exhibited a lipid carbonyl shoulder were quantified.

**Hour**	**Total Cells**	**Cells with shoulder**	**Percent of cells with shoulder**	**Total LDs**	**LDs with shoulder**	**Percent of LDs with shoulder**	**Total spectra**	**Spectra with shoulder**	**Percent Spectra with shoulder**
**1**	5	0	0%	14	0	0%	76	0	0%
**2**	3	0	0%	6	0	0%	61	0	0%
**3**	4	0	0%	12	0	0%	38	0	0%
**6**	5	0	0%	14	0	0%	76	0	0%
**9**	7	0	0%	26	0	0%	88	0	0%
**12**	16	3	19%	38	3	8%	100	19	19%
**15**	4	3	75%	7	4	57%	81	40	49%
**18**	5	5	100%	14	9	64%	88	51	58%
**21**	3	3	100%	6	2	33%	86	45	52%
**27**	4	4	100%	12	8	67%	92	55	60%
**30**	6	5	83%	19	8	42%	70	17	24%
**33**	4	2	50%	17	3	18%	86	32	37%
**36**	6	2	33%	20	4	20%	78	30	38%
**39**	3	3	100%	16	10	63%	93	54	58%
**42**	9	6	67%	40	28	70%	34	24	71%
**45**	8	5	63%	29	12	41%	58	18	31%
**51**	5	3	60%	21	4	20%	81	22	27%
**54**	5	2	40%	21	2	10%	81	29	36%
**57**	5	1	20%	24	2	8%	88	10	11%
**60**	7	2	29%	25	2	8%	93	8	9%
**63**	4	1	25%	16	1	6%	94	6	6%
**66**	7	3	43%	26	4	15%	82	9	11%
**69**	8	0	0%	33	0	0%	56	0	0%
**Total**	**133**	**53**	**40%**	**456**	**106**	**23%**	**1780**	**469**	**26%**

Table S3. Counts of 1734 cm^{-1} shoulder buildup throughout 70 hours of deuterated oleic acid (OA-d_{33}) feeding. Total number of cells, lipid droplets (LDs), and spectra that exhibited a lipid carbonyl shoulder were quantified.

Hour	Total Cells	Cells with shoulder	Percent of cells with shoulder	Total LDs	LDs with shoulder	Percent of LDs with shoulder	Total spectra	Spectra with shoulder	Percent Spectra with shoulder
**1**	11	0	0%	27	0	0%	48	0	0%
**2**	9	0	0%	24	0	0%	67	0	0%
**3**	4	0	0%	11	0	0%	78	0	0%
**6**	10	1	10%	15	1	7%	57	27	47%
**9**	6	0	0%	16	0	0%	80	0	0%
**12**	10	0	0%	27	0	0%	70	0	0%
**15**	6	1	17%	18	1	6%	59	1	2%
**19**	4	0	0%	11	0	0%	82	0	0%
**22**	3	1	33%	11	1	9%	90	3	3%
**27**	9	2	22%	24	2	8%	87	17	20%
**30**	6	1	17%	13	1	8%	97	8	8%
**33**	5	1	20%	22	1	5%	72	14	19%
**36**	6	1	17%	18	1	6%	80	1	1%
**39**	2	2	100%	6	2	33%	73	20	27%
**43**	11	1	9%	32	1	3%	114	14	12%
**46**	7	0	0%	16	0	0%	77	0	0%
**51**	8	0	0%	19	0	0%	123	0	0%
**54**	3	2	67%	12	2	17%	100	6	6%
**57**	6	2	33%	16	2	13%	89	21	24%
**60**	4	2	50%	10	2	20%	104	18	17%
**63**	4	1	25%	7	1	14%	92	4	4%
**67**	7	1	14%	26	1	4%	99	15	15%
**70**	7	1	14%	28	1	4%	61	14	23%
**Total**	**148**	**20**	**14%**	**409**	**20**	**5%**	**1899**	**183**	**10%**

REFERENCES

1. S. Iturbe-Rey, C. Maccali, M. Arrese, P. Aspichueta, C. P. Oliveira, R. E. Castro, A. Lapitz, L. Izquierdo-Sanchez, L. Bujanda, M. J. Perugorria, J. M. Banales, P. M. Rodrigues, Lipotoxicity-driven metabolic dysfunction-associated steatotic liver disease (MASLD). *Atherosclerosis* **400**, 119053 (2025).

2. K. Riazi, H. Azhari, J. H. Charette, F. E. Underwood, J. A. King, E. E. Afshar, M. G. Swain, S. E. Congly, G. G. Kaplan, A.-A. Shaheen, The prevalence and incidence of NAFLD worldwide: A systematic review and meta-analysis. *Lancet Gastroenterol. Hepatol.* **7**, 851–861 (2022).

3. R. T. Brookheart, C. I. Michel, J. E. Schaffer, As a matter of fat. *Cell Metab.* **10**, 9–12 (2009).

4. J. A. Olzmann, P. Carvalho, Dynamics and functions of lipid droplets. *Nat. Rev. Mol. Cell Biol.* **20**, 137–155 (2019).

5. A. J. Mathiowetz, J. A. Olzmann, Lipid droplets and cellular lipid flux. *Nat. Cell Biol.* **26**, 331–345 (2024).

6. M. Alves-Bezerra, D. E. Cohen, Triglyceride metabolism in the liver. *Compr. Physiol.* **8**, 1–8 (2017).

7. B. Bhatt-Wessel, T. W. Jordan, J. H. Miller, L. Peng, Role of DGAT enzymes in triacylglycerol metabolism. *Arch. Biochem. Biophys.* **655**, 1–11 (2018).

8. K. Takeuchi, K. Reue, Biochemistry, physiology, and genetics of GPAT, AGPAT, and lipin enzymes in triglyceride synthesis. *Am. J. Physiol. Endocrinol. Metab.* **296**, E1195–E1209 (2009).

9. L. Brohée, J. Crémer, A. Colige, C. Deroanne, Lipin-1, a versatile regulator of lipid homeostasis, is a potential target for fighting cancer. *Int. J. Mol. Sci.* **22**, 4419 (2021).

10. H. Wang, M. Becuwe, B. E. Housden, C. Chitraju, A. J. Porras, M. M. Graham, X. N. Liu, A. R. Thiam, D. B. Savage, A. K. Agarwal, A. Garg, M.-J. Olarte, Q. Lin, F. Fröhlich, H. K. Hannibal-

Bach, S. Upadhyayula, N. Perrimon, T. Kirchhausen, C. S. Ejsing, T. C. Walther, R. V. Farese Jr., Seipin is required for converting nascent to mature lipid droplets. *eLife* **5**, e16582 (2016).

11. H. B. Castillo, S. O. Shuster, L. H. Tarekegn, C. M. Davis, Oleic acid differentially affects lipid droplet storage of de novo synthesized lipids in hepatocytes and adipocytes. *Chem. Commun.* **60**, 3138–3141 (2024).

12. Y. Wei, D. Wang, F. Topczewski, M. J. Pagliassotti, Saturated fatty acids induce endoplasmic reticulum stress and apoptosis independently of ceramide in liver cells. *Am. J. Physiol. Endocrinol. Metab.* **291**, E275–E281 (2006).

13. M. Ricchi, M. R. Odoardi, L. Carulli, C. Anzivino, S. Ballestri, A. Pinetti, L. I. Fantoni, F. Marra, M. Bertolotti, S. Banni, A. Lonardo, N. Carulli, P. Loria, Differential effect of oleic and palmitic acid on lipid accumulation and apoptosis in cultured hepatocytes. *J. Gastroenterol. Hepatol.* **24**, 830–840 (2009).

14. A. Eynaudi, F. Díaz-Castro, J. C. Bórquez, R. Bravo-Sagua, V. Parra, R. Troncoso, Differential effects of oleic and palmitic acids on lipid droplet-mitochondria interaction in the hepatic cell line HepG2. *Front. Nutr.* **8**, 775382 (2021).

15. P. N. Paramitha, R. Zakaria, A. Maryani, Y. Kusaka, B. B. Andriana, K. Hashimoto, H. Nakazawa, S. Kato, H. Sato, Raman study on lipid droplets in hepatic cells co-cultured with fatty acids. *Int. J. Mol. Sci.* **22**, 7378 (2021).

16. L. L. Listenberger, X. Han, S. E. Lewis, S. Cases, R. V. Farese, D. S. Ory, J. E. Schaffer, Triglyceride accumulation protects against fatty acid-induced lipotoxicity. *Proc. Natl. Acad. Sci. U.S.A.* **100**, 3077–3082 (2003).

17. C. Moliterni, F. Vari, E. Schifano, S. Tacconi, E. Stanca, M. Friuli, S. Longo, M. Conte, S. Salvioli, D. Gnocchi, A. Mazzocca, D. Uccelletti, D. Vergara, L. Dini, A. M. Giudetti, Lipotoxicity of palmitic acid is associated with DGAT1 downregulation and abolished by PPARα activation in liver cells. *J. Lipid Res.* **65**, 100692 (2024).

18. S. O. Shuster, M. J. Burke, C. M. Davis, Spatiotemporal heterogeneity of de novo lipogenesis in fixed and living single cells. *J. Phys. Chem. B* **127**, 2918–2926 (2023).

19. N. Alsabeeh, B. Chausse, P. A. Kakimoto, A. J. Kowaltowski, O. Shirihai, Cell culture models of fatty acid overload: Problems and solutions. *Biochim. Biophys. Acta Mol. Cell Biol. Lipids* **1863**, 143–151 (2018).

20. A. M. Kleinfeld, D. Prothro, D. L. Brown, R. C. Davis, G. V. Richieri, A. DeMaria, Increases in serum unbound free fatty acid levels following coronary angioplasty. *Am. J. Cardiol.* **78**, 1350–1354 (1996).

21. C. Park, J. M. Lim, S.-C. Hong, M. Cho, Monitoring the synthesis of neutral lipids in lipid droplets of living human cancer cells using two-color infrared photothermal microscopy. *Chem. Sci.* **15**, 1237–1247 (2024).

22. M. Portaccio, B. Faramarzi, M. Lepore, Probing biochemical differences in lipid components of human cells by means of ATR-FTIR spectroscopy. *Biophysica* **3**, 524–538 (2023).

23. X. Chen, K. Chen, J. Hu, Y. Dong, M. Zheng, J. Jiang, Q. Hu, W. Zhang, Palmitic acid induces lipid droplet accumulation and senescence in nucleus pulposus cells via ER-stress pathway. *Commun. Biol.* **7**, 539 (2024).

24. M. F. Renne, H. Hariri, Lipid droplet-organelle contact sites as hubs for fatty acid metabolism, trafficking, and metabolic channeling. *Front. Cell Dev. Biol.* **9**, 726261 (2021).

25. L. Scorrano, M. A. De Matteis, S. Emr, F. Giordano, G. Hajnóczky, B. Kornmann, L. L. Lackner, T. P. Levine, L. Pellegrini, K. Reinisch, R. Rizzuto, T. Simmen, H. Stenmark, C. Ungermann, M. Schuldiner, Coming together to define membrane contact sites. *Nat. Commun.* **10**, 1287 (2019).

26. A. Pérez-Martí, S. Ramakrishnan, J. Li, A. Dugourd, M. R. Molenaar, L. R. De La Motte, K. Grand, A. Mansouri, M. Parisot, S. S. Lienkamp, J. Saez-Rodriguez, M. Simons, Reducing lipid bilayer stress by monounsaturated fatty acids protects renal proximal tubules in diabetes. *eLife* **11**, e74391 (2022).

27. M. Piccolis, L. M. Bond, M. Kampmann, P. Pulimeno, C. Chitraju, C. B. K. Jayson, L. P. Vaites, S. Boland, Z. W. Lai, K. R. Gabriel, S. D. Elliott, J. A. Paulo, J. W. Harper, J. S. Weissman, T. C. Walther, R. V. Farese Jr., Probing the global cellular responses to lipotoxicity caused by saturated fatty acids. *Mol. Cell* **74**, 32–44.e8 (2019).

28. A. Allerhand, P. von R. Schleyer, Solvent effects in infrared spectroscopic studies of hydrogen bonding. *J. Am. Chem. Soc.* **85**, 371–380 (1963).

29. K. Pal, I. Samanta, R. K. Gupta, D. Goswami, A. L. Koner, Deciphering micro-polarity inside the endoplasmic reticulum using a two-photon active solvatofluorochromic probe. *Chem. Commun.* **54**, 10590–10593 (2018).

30. B. Stuart, *Infrared Spectroscopy: Fundamentals and Applications* (Wiley, 2004).

31. A. Akoumi, T. Haffar, M. Mousterji, R. S. Kiss, N. Bousette, Palmitate mediated diacylglycerol accumulation causes endoplasmic reticulum stress, Plin2 degradation, and cell death in H9C2 cardiomyoblasts. *Exp. Cell Res.* **354**, 85–94 (2017).

32. F. D. Gunstone, J. L. Harwood, J. L. Harwood, *The Lipid Handbook with CD-ROM* (CRC Press, ed. 3, 2007).

33. G. H. Charbonnet, W. S. Singleton, Thermal properties of fats and oils. *J. Am. Oil Chem. Soc.* **24**, 140–142 (1947).

34. F. O. Cedeño, M. M. Prieto, A. Espina, J. R. García, Measurements of temperature and melting heat of some pure fatty acids and their binary and ternary mixtures by differential scanning calorimetry. *Thermochim. Acta* **369**, 39–50 (2001).

35. R. N. A. H. Lewis, R. N. McElhaney, Membrane lipid phase transitions and phase organization studied by Fourier transform infrared spectroscopy. *Biochim. Biophys. Acta* **1828**, 2347–2358 (2013).

36. M. Pilarczyk, T. P. Wrobel, M. Baranska, A. Kaczor, Correlation of monomer structures of tripalmitin with the spectroscopic fingerprint of polymorphs: Infrared, Raman, and DFT study. *J. Raman Spectrosc.* **43**, 1515–1522 (2012).

37. M. Uematsu, T. Shimizu, Raman microscopy-based quantification of the physical properties of intracellular lipids. *Commun. Biol.* **4**, 1176 (2021).

38. M. V. Fraile, B. Patrón-Gallardo, G. López-Rodríguez, P. Carmona, FT-IR study of multilamellar lipid dispersions containing cholesteryl linoleate and dipalmitoylphosphatidylcholine. *Chem. Phys. Lipids* **97**, 119–128 (1999).

39. L. Pellegrino, G. Tyagi, E. S. J. Robles, J. T. Cabral, Phase behaviour of model triglyceride ternary blends: Triolein, tripalmitin and tristearin. *Phys. Chem. Chem. Phys.* **24**, 29413–29422 (2022).

40. T. Sieling, T. Petersen, T. Alpers, J. Christoffers, T. Klüner, I. Brand, CD stretching modes are sensitive to the microenvironment in ionic liquids. *Chemistry* **27**, 17808–17817 (2021).

41. E. Tyrode, J. Hedberg, A comparative study of the CD and CH stretching spectral regions of typical surfactants systems using VSFS: Orientation analysis of the terminal CH_3 and CD_3 groups. *J. Phys. Chem. C* **116**, 1080–1091 (2012).

42. T. L. Brown, Infrared intensities and molecular structure. *Chem. Rev.* **58**, 581–608 (1958).

43. R. Pei, J. Zhang, J. Tan, Y. Luo, S. Ye, Fermi resonance of the N-D stretching mode probing the local hydrogen-bonding environment in proteins. *J. Phys. Chem. B* **128**, 5658–5666 (2024).

44. K. Tian, S. Ye, Fermi resonant interaction of the tailed methyl groups of Langmuir monolayer at the air/water interface during phase transition. *J. Phys. Chem. C* **119**, 25394–25400 (2015).

45. D. J. Moore, M. E. Rerek, Insights into the molecular organization of lipids in the skin barrier from infrared spectroscopy studies of stratum corneum lipid models. *Acta Derm. Venereol. Suppl.* **80**, 16–22 (2000).

46. M. F. Renne, R. Ernst, Membrane homeostasis beyond fluidity: Control of membrane compressibility. *Trends Biochem. Sci.* **48**, 963–977 (2023).

47. Z. Getahun, C.-Y. Huang, T. Wang, B. De León, W. F. DeGrado, F. Gai, Using nitrile-derivatized amino acids as infrared probes of local environment. *J. Am. Chem. Soc.* **125**, 405–411 (2003).

48. C. G. Bazewicz, M. T. Liskov, K. J. Hines, S. H. Brewer, Sensitive, site-specific, and stable vibrational probe of local protein environments: 4-Azidomethyl-l-phenylalanine. *J. Phys. Chem. B* **117**, 8987–8993 (2013).

49. L. Shi, X. Liu, L. Shi, H. T. Stinson, J. Rowlette, L. J. Kahl, C. R. Evans, C. Zheng, L. E. P. Dietrich, W. Min, Mid-infrared metabolic imaging with vibrational probes. *Nat. Methods* **17**, 844–851 (2020).

50. Y. Bai, C. M. Camargo, S. M. K. Glasauer, R. Gifford, X. Tian, A. P. Longhini, K. S. Kosik, Single-cell mapping of lipid metabolites using an infrared probe in human-derived model systems. *Nat. Commun.* **15**, 350 (2024).

51. J. H. Ahn, M. H. Kim, H. J. Kwon, S. Y. Choi, H. Y. Kwon, Protective effects of oleic acid against palmitic acid-induced apoptosis in pancreatic AR42J cells and its mechanisms. *Korean J. Physiol. Pharmacol.* **17**, 43–50 (2013).

52. J. A. Ontko, L. W. Perrin, L. S. Horne, Isolation of hepatocellular lipid droplets: The separation of distinct subpopulations. *J. Lipid Res.* **27**, 1097–1103 (1990).

53. J. P. Layerenza, P. González, M. M. García de Bravo, M. P. Polo, M. S. Sisti, A. Ves-Losada, Nuclear lipid droplets: A novel nuclear domain. *Biochim. Biophys. Acta* **1831**, 327–340 (2013).

54. C. Dumesnil, L. Vanharanta, X. Prasanna, M. Omrane, M. Carpentier, A. Bhapkar, G. Enkavi, V. T. Salo, I. Vattulainen, E. Ikonen, A. R. Thiam, Cholesterol esters form supercooled lipid droplets whose nucleation is facilitated by triacylglycerols. *Nat. Commun.* **14**, 915 (2023).

55. M.-C. Popescu, C. Vasile, D. Filip, D. Macocinschi, Gh. Singurel, Characterization by Fourier transform infrared spectroscopy of polyethylene adipate/cholesteryl palmitate blends. *J. Appl. Polym. Sci.* **94**, 1156–1163 (2004).

56. S. Hong, S. Rhee, K. O. Jung, In vivo molecular and single cell imaging. *BMB Rep.* **55**, 267–274 (2022).

57. X. Li, Y. Li, M. Jiang, W. Wu, S. He, C. Chen, Z. Qin, B. Z. Tang, H. Y. Mak, J. Y. Qu, Quantitative imaging of lipid synthesis and lipolysis dynamics in Caenorhabditis elegans by stimulated Raman scattering microscopy. *Anal. Chem.* **91**, 2279–2287 (2019).

58. D. Zhang, M. N. Slipchenko, J.-X. Cheng, Highly sensitive vibrational imaging by femtosecond pulse stimulated Raman loss. *J. Phys. Chem. Lett.* **2**, 1248–1253 (2011).

59. Y. Tan, J. Li, G. Zhao, K.-C. Huang, H. Cardenas, Y. Wang, D. Matei, J.-X. Cheng, Metabolic reprogramming from glycolysis to fatty acid uptake and beta-oxidation in platinum-resistant cancer cells. *Nat. Commun.* **13**, 4554 (2022).

60. D. Fu, Y. Yu, A. Folick, E. Currie, R. V. Farese, T.-H. Tsai, X. S. Xie, M. C. Wang, In vivo metabolic fingerprinting of neutral lipids with hyperspectral stimulated Raman scattering microscopy. *J. Am. Chem. Soc.* **136**, 8820–8828 (2014).

61. Y. Shen, Z. Zhao, L. Zhang, L. Shi, S. Shahriar, R. B. Chan, G. Di Paolo, W. Min, Metabolic activity induces membrane phase separation in endoplasmic reticulum. *Proc. Natl. Acad. Sci. U.S.A.* **114**, 13394–13399 (2017).

62. J. R. Ferraro, K. Nakamoto, C. Brown, “Basic theory,” in *Introductory Raman Spectroscopy* (Elsevier, ed. 2, 2003), pp. 1–91.

63. X. Teng, T. Xia, J. Yin, M. Li, J. Ao, G. Ding, C. V. Prabhu Dessai, A. M. Isac, D. Matei, H. He, J.-X. Cheng, Mid-infrared photothermal imaging of fatty acid desaturation reaction in cancer cells. bioRxiv 662881 [Preprint] (2025). https://doi.org/10.1101/2025.07.02.662881.

64. T. O. Eichmann, A. Lass, DAG tales: The multiple faces of diacylglycerol—stereochemistry, metabolism, and signaling. *Cell. Mol. Life Sci.* **72**, 3931–3952 (2015).

65. M. C. Petersen, G. I. Shulman, Roles of diacylglycerols and ceramides in hepatic insulin resistance. *Trends Pharmacol. Sci.* **38**, 649–665 (2017).

66. K. Lipke, A. Kubis-Kubiak, A. Piwowar, Molecular mechanism of lipotoxicity as an interesting aspect in the development of pathological states—Current view of knowledge. *Cells* **11**, 844 (2022).

67. N. G. Løvsletten, H. Vu, C. Skagen, J. Lund, E. T. Kase, G. H. Thoresen, V. A. Zammit, A. C. Rustan, Treatment of human skeletal muscle cells with inhibitors of diacylglycerol acyltransferases 1 and 2 to explore isozyme-specific roles on lipid metabolism. *Sci. Rep.* **10**, 238 (2020).

68. M. Longo, E. Paolini, P. Di Benedetto, E. Tomassini, M. Meroni, P. Dongiovanni, DGAT1 and DGAT2 inhibitors for metabolic dysfunction-associated steatotic liver disease (MASLD) management: Benefits for their single or combined application. *Int. J. Mol. Sci.* **25**, 9074 (2024).

69. X. Liu, R. M. Green, Endoplasmic reticulum stress and liver diseases. *Liver Res.* **3**, 55–64 (2019).

70. F. Sarnyai, A. Somogyi, Z. Gór-Nagy, V. Zámbó, P. Szelényi, J. Mátyási, L. Simon-Szabó, É. Kereszturi, B. Tóth, M. Csala, Effect of cis- and trans-monounsaturated fatty acids on palmitate toxicity and on palmitate-induced accumulation of ceramides and diglycerides. *Int. J. Mol. Sci.* **21**, 2626 (2020).

71. K. Halbleib, K. Pesek, R. Covino, H. F. Hofbauer, D. Wunnicke, I. Hänelt, G. Hummer, R. Ernst, Activation of the unfolded protein response by lipid bilayer stress. *Mol. Cell* **67**, 673–684.e8 (2017).

72. F. Wilfling, H. Wang, J. T. Haas, N. Krahmer, T. J. Gould, A. Uchida, J.-X. Cheng, M. Graham, R. Christiano, F. Fröhlich, X. Liu, K. K. Buhman, R. A. Coleman, J. Bewersdorf, R. V. Farese,

T. C. Walther, Triacylglycerol synthesis enzymes mediate lipid droplet growth by relocalizing from the ER to lipid droplets. *Dev. Cell* **24**, 384–399 (2013).

73. V. Choudhary, G. Golani, A. S. Joshi, S. Cottier, R. Schneiter, W. A. Prinz, M. M. Kozlov, Architecture of lipid droplets in endoplasmic reticulum is determined by phospholipid intrinsic curvature. *Curr. Biol.* **28**, 915–926.e9 (2018).

74. R. A. Petazzi, A. A. Koikkarah, N. D. Tischler, S. Chiantia, Detection of envelope glycoprotein assembly from old-world hantaviruses in the Golgi apparatus of living cells. *J. Virol.* **95**, e01238-20 (2021).

75. J. Schindelin, I. Arganda-Carreras, E. Frise, V. Kaynig, M. Longair, T. Pietzsch, S. Preibisch, C. Rueden, S. Saalfeld, B. Schmid, J.-Y. Tinevez, D. J. White, V. Hartenstein, K. Eliceiri, P. Tomancak, A. Cardona, Fiji: An open-source platform for biological-image analysis. *Nat. Methods* **9**, 676–682 (2012).

76. G. Van Rossum, F. L. Drake, *Python 3 Reference Manual* (CreateSpace, 2009).

77. T. Okuno, T. Yokomizo, "Basic techniques for lipid extraction from tissues and cells," in *Bioactive Lipid Mediators: Current Reviews and Protocols* (Springer Japan, 2015), pp. 331–336. https://doi.org/10.1007/978-4-431-55669-5_23.

78. J. Chung, X. Wu, T. J. Lambert, Z. W. Lai, T. C. Walther, R. V. Farese Jr., LDAF1 and seipin form a lipid droplet assembly complex. *Dev. Cell* **51**, 551–563.e7 (2019).

79. S. C. Hsi, A. P. Tulloch, H. H. Mantsch, D. G. Cameron, A vibrational study of the CD2 stretching bands of selectively deuterated palmitic and stearic acids. *Chem. Phys. Lipids* **31**, 97–103 (1982).

80. A. Filopoulou, S. Vlachou, S. C. Boyatzis, Fatty acids and their metal salts: A review of their infrared spectra in light of their presence in cultural heritage. *Molecules* **26**, 6005 (2021).
